# Deformable registration and generative modelling of aortic anatomies by auto-decoders and neural ODEs

**DOI:** 10.1038/s44341-025-00029-z

**Published:** 2025-12-03

**Authors:** Riccardo Tenderini, Luca Pegolotti, Fanwei Kong, Stefano Pagani, Francesco Regazzoni, Alison L. Marsden, Simone Deparis

**Affiliations:** 1 Institute of Mathematics, EPFL, Lausanne, Switzerland; 2 Department of Bioengineering, Stanford University, Stanford, CA USA; 3 Department of Pediatrics, Stanford University, Stanford, CA USA; 4 Institute for Computational and Mathematical Engineering, Stanford University, Stanford, CA USA; 5 Department of Mechanical Engineering and Materials Science, Washington University, St. Louis, MO USA; 6 MOX – Department of Mathematics, Politecnico di Milano, Milan, Italy; 7 Cardiovascular Institute, Stanford University, Stanford, CA USA

**Keywords:** Machine learning, Applied physics, Fluid dynamics

## Abstract

Accurate registration of vascular shapes is essential for comparing anatomical geometries, extracting reliable measurements, and generating realistic models in cardiovascular research. Conventional surface registration methods often face limitations in efficiency, scalability, and generalization across shape cohorts. In this work, we present AD–SVFD, a deep learning framework that simultaneously performs deformable registration of vascular geometries to a pre–defined reference anatomy and enables the synthesis of new shapes. AD–SVFD represents each geometry as a point cloud and models ambient deformations as solutions at unit time of ordinary differential equations (ODEs), whose time–independent right–hand sides are parameterized by neural networks. Registration is optimized by minimizing the Chamfer distance between deformed and reference geometries, while shape generation is achieved by integrating the ODE backward in time from sampled latent codes. A distinctive auto–decoder architecture associates each anatomy with a low–dimensional embedding, jointly optimized with the network parameters during training, and fine–tuned at inference, reducing computational overhead. Numerical experiments on healthy aortic anatomies demonstrate the capability of AD–SVFD to yield accurate approximations at competitive computational costs. Compared to existing approaches, our model offers an efficient, unified framework for processing multiple shapes and robustly generating plausible geometries.

## Introduction

Over the last two decades, the deformable registration of three-dimensional images has become increasingly important in a wide number of computer graphics and computer vision applications. In broad terms, the deformable—or non-rigid—registration problem consists of aligning and locating different shapes within a shared coordinate system, to enable meaningful comparisons and analyses^1–3^. Besides industrial and engineering applications, deformable registration nowadays plays a crucial role in several medical imaging tasks, such as multimodal image fusion, organ atlas creation, and monitoring of disease progression^4,5^. Unlike rigid registration, which involves only global scaling, rotations, and translations, deformable registration must estimate complex, localized deformation fields that account for natural anatomical variability. This challenge is enhanced by the presence of noise, outliers, and partial overlaps, which are very common in clinical data. Furthermore, exact point-to-point correspondences between different anatomies are rarely available in practice, which requires the adoption of alternative metrics to evaluate data adherence.

The challenge of developing efficient, reliable, and computationally tractable registration methods is of paramount importance for improving medical imaging workflows, healthcare technologies, and patient care. Manual alignment of images in subject-specific clinical contexts is often infeasible or impractical, due to the complexity and variability of biological structures, as well as to the differences in imaging modalities and acquisition times. To address this limitation, several automatic registration approaches have been developed. Among the most widely employed ones, we can mention DARTEL^3^, Diffeomorphic Demons^6^, and LDDMM^7–9^. Notably, all these methods share remarkable robustness characteristics, since they are based on a deformation of the ambient space, which is guaranteed to be smooth, differentiable, invertible, and topology preserving.

While traditional image and shape registration approaches can yield extremely accurate results, they nonetheless entail non-negligible computational costs that may hinder their use in real-time clinical practice. To mitigate this issue and improve the overall performance, deep learning (DL) techniques have been exploited in various ways. A non-exhaustive list of the most popular state-of-the-art DL-based registration methods includes the probabilistic models developed in refs. ^10,11^, *Voxelmorph*^12^, *Smooth Shells*^13^, *Neuromorph*^14^, *Cyclemorph*^15^, *Diffusemorph*^16^ and *Transmorph*^17^. We refer to refs. ^5,18–20^ for comprehensive literature reviews on the topic.

In our study, we are specifically interested in the registration of vascular surfaces. The latter can be seamlessly extracted from volumetric data, acquired through traditional imaging modalities, such as CT-scans or MRI. Furthermore, novel techniques like photoacoustic scanning^21–23^ are rapidly gaining traction in clinical practice, since they provide a low-cost radiation-free alternative, particularly well-suited for superficial vascular anatomies, located up to 15 mm beneath the skin. A review of the classical techniques for surface registration can be found in ref. ^24^. In this scenario, DL-based approaches can be subdivided into two major groups, depending on how surfaces are represented. On the one hand, we have methods that treat shapes as 3D point clouds^25^, such as the ones introduced in refs. ^26–28^. On the other hand, instead, there exist several methods that represent 3D geometries by means of Deep Implicit Functions—namely continuous signed distance functions, expressed through neural networks^29–31^—such as the ones presented in refs. ^32,33^. Notably, the models described in refs. ^27,32,33^ encapsulate learnable latent shape representations, which enable the simultaneous registration of multiple geometries to a common reference, as well as their use as generative AI tools.

**Fig. 1:**
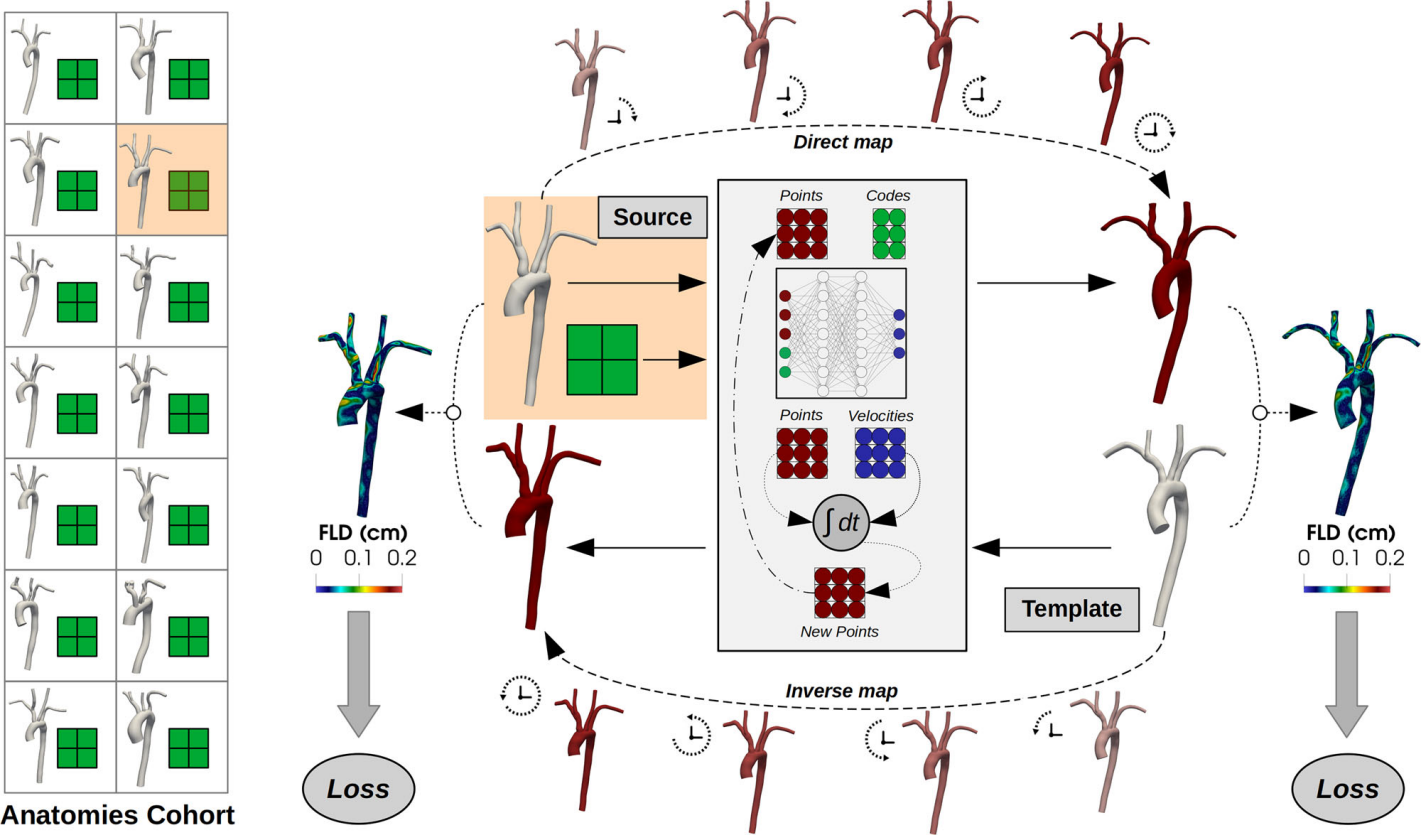
General structure of the AD-SVFD model. The proposed approach leverages deep learning techniques to perform the diffeomorphic registration of vascular anatomies to a reference. Invertible ambient space deformations are modeled as solutions at unit time of ODEs, whose right-hand sides are parametrized by neural networks. The source and template geometries, represented as point clouds, are provided as input to AD-SVFD. The direct (top part of the image) and inverse (bottom part of the image) transforms are obtained by integrating the flow equations forward and backward in time, respectively. Geodesic paths can be visualized by morphing the input shapes at intermediate stages during the ODE integration. Generalization capabilities are enabled by associating each source shape with a trainable latent code (in green). The baseline model is optimized by minimizing the Chamfer distance (CD) between the mapped and the target geometries. Pointwise errors are quantified through the forward local distance (FLD), expressed in cm, namely the distance of each point in the mapped geometry from the closest one in the target.

In this work, we present a DL-based model for the deformable registration and synthetic generation of vascular anatomies, named AD-SVFD (*Auto-Decoder Stationary Vector Field Diffeomorphism*). The general structure of AD-SVFD, reported in Fig. 1, is inspired by the models introduced by Amor et al. in ref. ^28^ (*ResNet-LDDMM*), by Kong et al. in ref. ^33^ (*SDF4CHD*), and by Croquet et al. in ref. ^27^. Analogously to refs. ^27,28^, AD-SVFD treats geometries as three-dimensional point clouds and employs ad hoc data attachment measures to compensate for the absence of ground-truth point-to-point correspondences. The vascular shapes registration is achieved by deforming the ambient space according to an optimizable diffeomorphic map. The latter is approximated as the solution at unit time of an ordinary differential equation (ODE), whose time-independent right-hand side, representing a velocity field, is expressed through a fully-connected artificial neural network (ANN) (*Neural ODE* paradigm^34^). Another major feature of AD-SVFD is its auto-decoder (AD) structure, introduced in a similar context in ref. ^31^ (*DeepSDF*) and then further exploited, e.g., in ref. ^33^. In fact, AD-SVFD enables the simultaneous registration of a cohort of source shapes to a pre-defined common reference by introducing low-dimensional learnable latent codes that are provided as input to the model and that condition its weights. As such, AD-SVFD configures as a self-conditional neural field^35^, since the conditioning variable is part of the model trainables. Compared to the more widely employed auto-encoders (AEs)^36–38^, that obtain latent input representations through a trainable encoding network, ADs entail faster and lighter optimization processes. Indeed, they roughly halve model complexity, at the cost of a cheap latent code inference procedure to be performed at the testing stage. Other than featuring improved generalization capabilities and favoring efficient weight sharing, implicit neural representations through latent codes also enable generative AI applications^39^. Indeed, synthetic anatomies can be crafted by drawing samples from empirical distributions, defined over the low-dimensional latent space, and by applying the associated inverse transforms to the reference geometry.

We remark that AD-SVFD introduces major methodological novelties compared to the approaches presented in refs. ^27,28,33^. First, the adoption of an auto-decoder structure distinguishes AD-SVFD from both ResNet-LDDMM, which does not employ latent codes, and ref. ^27^, which relies on a convolutional variational autoencoder. Second, unlike ResNet-LDDMM, AD-SVFD represents the diffeomorphic map through a stationary vector field parametrization embedded in a Neural ODE, rather than through a variable velocity field approximated via residual connections. Third, in contrast to SDF4CHD, AD-SVFD does not rely on signed distance functions (SDFs) for geometry representation, but operates directly on three-dimensional surface point clouds, which are better suited to vascular anatomies. Finally, unlike all three prior approaches, AD-SVFD incorporates a loss penalization on both the direct and inverse mappings, thereby favouring the learning of diffeomorphic maps with numerically stable inverses. It is worth noting, however, that AD-SVFD does not include the *Atlas* learning capabilities of SDF4CHD, hence requiring the availability of a pre-defined reference shape. We refer to the *Methods* section for a detailed discussion.

For clarity of presentation and to support the interpretation of the numerical results reported in the following section, we provide a brief description of the proposed methodology. Let ${\mathcal{T}}$ denote the template (or reference) geometry and let ${\{{{\mathcal{S}}}_{i}\}}_{i = 1}^{{N}^{s}}$ denote the cohort of available patient-specific vascular anatomies; the latter will be referred to as the *source cohort* in the following. In particular, ${\mathcal{T}}$ and ${{\mathcal{S}}}_{i}$ identify three-dimensional closed surfaces that are represented as weighted point clouds of the form:

1 $${\mathcal{T}}:= {\left\{\left({{\boldsymbol{x}}}_{j}^{{\rm {t}}},{w}_{j}^{{\rm {t}}}\right)\right\}}_{j = 1}^{{M}^{{\rm {t}}}};\qquad {{\mathcal{S}}}_{i}:= {\left\{\left({{\boldsymbol{x}}}_{i,j}^{{\rm {s}}},{w}_{i,j}^{{\rm {s}}}\right)\right\}}_{j = 1}^{{M}_{i}^{{\rm {s}}}}\quad {\rm{for}}\,i=1,\ldots ,{N}^{{\rm {s}}}\,.$$

Here, ${{\boldsymbol{x}}}_{j}^{t},{{\boldsymbol{x}}}_{i,j}^{s}\in {{\mathbb{R}}}^{3}$ are, respectively, the template and source points, and ${w}_{j}^{t},{w}_{i,j}^{s}\in {{\mathbb{R}}}^{+}$ are the associated weights, which add up to one. In general, the weights associated with isolated points in the cloud should be large, while those in regions of high local density should be lower. In this work, we construct the point clouds from available triangular surface meshes by selecting the cell centers as points and computing the weights as the corresponding (normalized) cell areas. To facilitate training, we perform a preliminary rigid registration of the source shapes to the template, based on the coherent point drift algorithm^40^, and we apply an anisotropic rescaling. In this way, we embed every geometry in the unit cube *Ω* ≔ [0, 1]^3^ and we can assume that ${{\boldsymbol{x}}}_{j}^{t},{{\boldsymbol{x}}}_{i,j}^{s}\in \Omega$ without loss of generality. It is worth remarking that a tailored template shape can be estimated from the set of available anatomies, as done e.g. in refs. ^33,41–43^. However, for simplicity, in this work, we simply select one patient-specific anatomy to serve as a reference.

In mathematical terms, our goal is to find a set of diffeomorphisms ${\{{\overrightarrow{\varphi }}_{i}\}}_{i = 1}^{{N}^{{\rm {s}}}}$ that solves the following minimization problem:

2 $$\left({\overrightarrow{\varphi }}_{1}^{* },\ldots ,{\overrightarrow{\varphi }}_{{N}^{s}}^{* }\right)=\mathop{{\rm{arg}}\,{\rm{min}}}\limits_{\left({\overrightarrow{\varphi }}_{1},\ldots ,{\overrightarrow{\varphi }}_{{N}^{s}}\right)}\frac{1}{{N}^{s}}\mathop{\sum }\limits_{i=1}^{{N}^{s}}\left({\mathcal{D}}\left({\overrightarrow{\varphi }}_{i}({{\mathcal{S}}}_{i}),{\mathcal{T}}\right)+{\mathcal{D}}\left({{\mathcal{S}}}_{i},{({\overrightarrow{\varphi }}_{i})}^{-1}({\mathcal{T}})\right)\right)\,,$$

where ${\mathcal{D}}:{{\mathbb{R}}}^{{M}_{1}\times 3}\times {{\mathbb{R}}}^{{M}_{2}\times 3}\to {{\mathbb{R}}}^{+}$ is some discrepancy measure between two three-dimensional point clouds of cardinalities ${M}_{1},{M}_{2}\in {\mathbb{N}}$. Hence, we want to learn a family of invertible ambient space deformations, whose elements allow to optimally (i) map the source shapes to the template via the direct transforms ${\{{\overrightarrow{\varphi }}_{i}^{* }\}}_{i = 1}^{{N}^{{\rm {s}}}}$ and (ii) map the template shape to the sources via the inverse transforms ${\{{({\overrightarrow{\varphi }}_{i}^{* })}^{-1}\}}_{i = 1}^{{N}^{{\rm {s}}}}$. As discussed before, the AD-SVFD model features an auto-decoder structure, through the use of low-dimensional latent codes ${\{{{\boldsymbol{z}}}_{i}\}}_{i = 1}^{{N}^{{\rm {s}}}},\,{{\boldsymbol{z}}}_{i}\in {{\mathbb{R}}}^{{N}_{z}}$, associated with the source shapes. The ambient space deformation associated to ${{\mathcal{S}}}_{i}$ can be expressed as ${\overrightarrow{\varphi }}_{i}({\boldsymbol{x}})=\overrightarrow{\varphi }({\boldsymbol{x}};{\mathbf{\Theta }},{{\boldsymbol{z}}}_{i})$, entirely encapsulating the input dependency into the shape code. Therefore, the optimization problem in Eq.(2) can be conveniently rewritten as follows: find ${{\mathbf{\Theta }}}^{* }\in {{\mathbb{R}}}^{{N}_{\Theta }},\,{{\boldsymbol{z}}}_{i}^{* }\in {{\mathbb{R}}}^{{N}_{z}}\,{\rm{for}}\,i=1,\ldots ,{N}_{s}$, such that

$${{\mathbf{\Theta }}}^{* },\,\left({{\boldsymbol{z}}}_{1}^{* },\ldots ,{{\boldsymbol{z}}}_{{N}^{{\rm {s}}}}^{* }\right)=\mathop{{\rm{arg}\,{\rm{min}}}}\limits_{{\mathbf{\Theta }},\,\left({{\boldsymbol{z}}}_{1},\ldots ,{{\boldsymbol{z}}}_{{N}^{s}}\right)}\frac{1}{{N}^{{\rm {s}}}}\mathop{\sum }\limits_{i=1}^{{N}^{{\rm {s}}}}{\mathcal{E}}\left({{\mathcal{S}}}_{i},{\mathcal{T}},\overrightarrow{\varphi }(\,\cdot \,;{\mathbf{\Theta }},{{\boldsymbol{z}}}_{i})\right)\,,$$

where ${\mathcal{E}}({\mathcal{S}},{\mathcal{T}},\overrightarrow{\phi }):= {\mathcal{D}}(\overrightarrow{\varphi }({\mathcal{S}}),{\mathcal{T}})+{\mathcal{D}}({\overrightarrow{\varphi }}^{-1}({\mathcal{T}}),{\mathcal{S}})$ denotes the bidirectional mapping error between two point clouds ${\mathcal{S}}$ and ${\mathcal{T}}$, through the diffeomorphism $\overrightarrow{\varphi }$. In this setting, the latent codes can be interpreted as low-dimensional embeddings of the source shapes, obtained through the application of an encoder-like operator ${\mathcal{P}}({\mathbf{\Theta }}):{{\mathbb{R}}}^{M\times 3}\to {{\mathbb{R}}}^{{N}_{z}}$, where $M\in {\mathbb{N}}$ denotes the point-cloud cardinality, such that ${{\boldsymbol{z}}}_{i}={\mathcal{P}}({{\mathcal{S}}}_{i};{\mathbf{\Theta }}),\,i=1,\ldots ,{N}_{s}$. We remark that in a standard AE setting ${\mathcal{P}}$ would be explicitly parametrized by a neural network (encoder) and jointly optimized with the decoder. Here, instead, it configures as a by-product of the small optimization problem on the shape code entries, and it is implicitly defined through the decoder.

Adopting the stationary vector field (SVF) parametrization of diffeomorphisms^3,44^ (as done in refs. ^27,33^), we exploit the Neural ODE paradigm ^34^ to express the map *φ*_*i*_ as the solution at unit time to the following learnable ODE:

3 $$\frac{\partial {\overrightarrow{\varphi }}_{i}({\boldsymbol{x}};t)}{\partial t}=\vec{v}\left({\vec{\varphi }}_{i}({\boldsymbol{x}};t),{\mathbf{\Theta }},{{\boldsymbol{z}}}_{i}\right)\quad {\rm{such}}\,{\rm{that}}\quad {\vec{\varphi }}_{i}({\boldsymbol{x}};0)={\boldsymbol{x}}\,,$$

where the vector ${\mathbf{\Theta }}\in {{\mathbb{R}}}^{{N}_{\Theta }}$ collects the trainable parameters of an ANN. As demonstrated in ref. ^45^, if the ANN that expresses the velocity field $\vec{v}$ is fully connected and features *ReLU* or *Leaky-ReLU* activation functions, then $\overrightarrow{v}$ is Lipschitz continuous and Eq. (3) admits a unique solution. Consequently, the inverse transform ${({\vec{\varphi }}_{i})}^{-1}$, which deforms the ambient space so as to overlap the template point cloud ${\mathcal{T}}$ to the source one ${{\mathcal{S}}}_{i}$, can be found by integrating Eq. (3) backward in time. In this work, we employed the first-order forward Euler and modified Euler schemes to numerically integrate the diffeomorphic flow equations forward and backward in time, respectively, considering *K* = 10 discrete time steps, as in ref. ^28^.

Our approach is developed under the assumption that all shapes share the same topology. Conversely, it is not possible to guarantee the existence (and uniqueness) of a diffeomorphic flow field that exactly deforms one into the other. In fact, non-rigid registration under topological variability remains an open challenge^46^.

To train the AD-SVFD model, we employ the following loss function:

4 $${\mathcal{L}}({\mathbf{\Theta }},{\boldsymbol{Z}}):=\frac{1}{{N}^{{\rm {s}}}}\mathop{\sum }\limits_{i=1}^{{N}^{{\rm {s}}}}\left({\mathcal{E}}\left({{\mathcal{S}}}_{i},{\mathcal{T}};\vec{\varphi }(\cdot ;{\mathbf{\Theta }},{{\boldsymbol{z}}}_{i})\right)\right)+{w}_{z}{\parallel {\boldsymbol{Z}}\parallel }_{2}^{2}+{w}_{\Theta }{\parallel {\mathbf{\Theta }}\parallel }_{2}^{2}+{w}_{v}\,{{\mathcal{L}}}_{{\rm{reg}}}({\mathbf{\Theta }})\,,$$

where ${w}_{z},{w}_{\Theta },{w}_{v}\in {{\mathbb{R}}}^{+}$ are scalar weight factors, ${\boldsymbol{Z}}\in {{\mathbb{R}}}^{{N}_{z}\times {N}_{s}}$ is a matrix collecting the shape codes associated with the *N*_s_ training shapes, and ${{\mathcal{L}}}_{{\rm{reg}}}$ is a regularization term that constrains the velocity field learned by the ANN. In the numerical experiments, we explore multiple alternatives for the data attachment measure ${\mathcal{D}}$ that appears in the definition of the bidirectional mapping error ${\mathcal{E}}$. Specifically, we consider the Chamfer distance (CD)^47^, the point-to-plane Chamfer distance (PCD)^48^, the Chamfer distance endowed with a penalization on the normals’ orientation scaled by the factor ${w}_{n}\in {{\mathbb{R}}}^{+}$ (denoted as NCD), and the debiased Sinkhorn divergence (SD)^49^. Furthermore, we exploit the availability of weights (see Eq. (1)) to derive data adherence measures that should be better able to deal with unevenly distributed point clouds. The training procedure is carried out with the *Adam* optimizer^50^, considering *E* = 500 epochs, a batch size *B* = 8, and setting the same learning rate $\lambda \in {{\mathbb{R}}}^{+}$ to update the ANN parameters and the shape codes. At each epoch, sub-clouds made of *M* = 2000 points are adaptively sampled to limit computational efforts and memory requirements. More details on both the data attachment measures and the training pipeline are provided in the “Methods” section. During testing, we can combine *Adam* with higher-order memory-intensive methods, such as L-BFGS^51^, since only the latent code entries have to be optimized. Specifically, we first run 100 epochs using *Adam*, and then we fine-tune the predictions using L-BFGS for 10 epochs. Additionally, preliminary numerical results suggested using a learning rate 50 times larger than the one employed for training with *Adam*, as this facilitates and speeds up convergence.

## Results

**Fig. 2:**
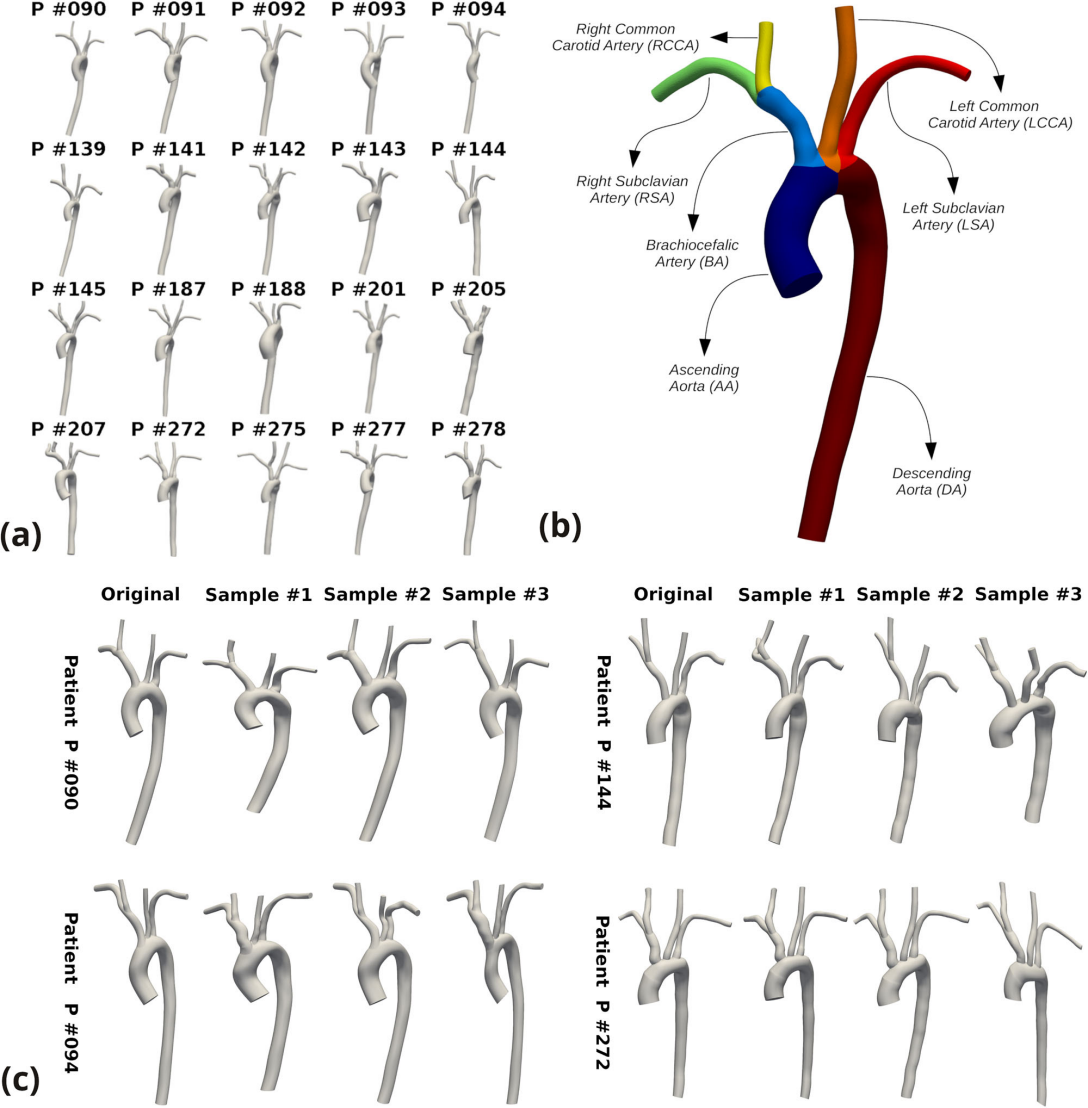
Healthy aortic shapes dataset overview. In particular: **a** original dataset of patient-specific anatomies, **b** topology of the considered geometries, with nomenclature of the different branches, **c** original shape and three synthetic samples, generated by deforming four anatomies with the implemented data augmentation pipeline.

We present the numerical experiments conducted on the AD-SVFD model and briefly discuss the obtained results. All tests have been performed starting from a dataset containing 20 healthy aortic anatomies, which have been segmented from medical images (CT-scans and MRIs) using *SimVascular*^52^ (see Fig. 2a) and are publicly available in the *Vascular Model Repository*^53^. As depicted in Fig. 2b, we underline that all the geometries share the same topology, which comprises the aortic vessel (ascending chunk (AA) and descending chunk (DA)), the brachiocefalic artery (BA), the left and right subclavian arteries (LSA, RSA), and the left and right common carotid arteries (LCCA, RCCA). To generate weighted point cloud representations of the shapes, we created volumetric tetrahedral computational meshes and extracted triangulations of the external surfaces. This allowed us to choose the surface cell centers as the cloud points, and the surface cell areas as the associated weights (see Eq. (1)).

The number of available anatomies is evidently too low to train a DL-based model, whose performances drastically depend on the amount of data at disposal. Therefore, we implemented an ad hoc data augmentation pipeline, based on Coherent Point Drift (CPD) rigid registration^40^ and thin-plate spline (TPS) interpolation^54^. We refer to Supplementary Notes 1 and 2 for a detailed description. This allowed us to assemble a dataset made of 902 anatomies, out of which 882 have been artificially generated. A few synthetic anatomies are reported in Fig. 2c. We perform a train-test splitting, reserving 38 shapes solely for testing. In particular, 2 of the test geometries belong to the original dataset, and their corresponding augmented versions are not taken into account for training; the remaining 36 test geometries are instead augmented versions of the 18 original anatomies included in the training dataset (2 augmented shapes per patient). Except for the cross-validation procedure, all the numerical tests are carried out considering the same training and testing datasets. They have been obtained by reserving patients *P#093* and *P#278* for testing, which results in employing 780 geometries for training. We remark that patient *P#091* serves as a reference in all test cases.

Most hyperparameters of the ANN model have been calibrated in a simplified single shape-to-shape registration scenario, using the Tree-structured Parzen estimator (TPE) Bayesian algorithm^55,56^. We refer to Supplementary Note 3 for a complete list of the hyperparameters and for a detailed description of the tuning procedure. Besides dictating the specifics of the ANN model architecture, the calibration results suggested to set the learning rate *λ*_*Θ*_ = *λ*_*z*_ = *λ* = 10^−3^, and the loss weights *w*_*v*_ = 10^−4^ and *w*_*z*_ = 10^−3^ (see Eq. (4)). Unless differently specified, the loss is computed considering the standard (i.e. not weighted) CD as a data attachment measure. The model accuracy is quantified through the forward and backward local distances (FLD and BLD), expressed in cm. The former identifies the distance of each point in the mapped geometry from the closest one in the target, while the latter is the distance of each point in the target from the closest one in the mapped geometry.

All computations were performed on the Sherlock cluster at *Stanford University*, employing an AMD 7502P processor (32 cores), 256 GB RAM, HDR InfiniBand interconnect, and a single NVIDIA GeForce RTX 2080 Ti GPU. The reported average testing times were obtained on the Kuma cluster at *EPFL*, considering a single NVIDIA H100 SXM5 GPUs, 94 GB RAM (HBM2e), memory bandwidth of 2.4 TB/s, Interconnected with NVLink, 900 GB/s bandwidth. We note that the exact reproducibility of the results cannot be guaranteed, owing to the use of non-deterministic algorithms provided by the *PyTorch* library to enhance efficiency.

### Test 1: Latent shape codes

We investigate the effect of shape codes on the AD-SVFD model results, focusing in particular on the latent space dimension *N*_*z*_. We point out that the training errors are computed only on the 18 original shapes.

**Fig. 3:**
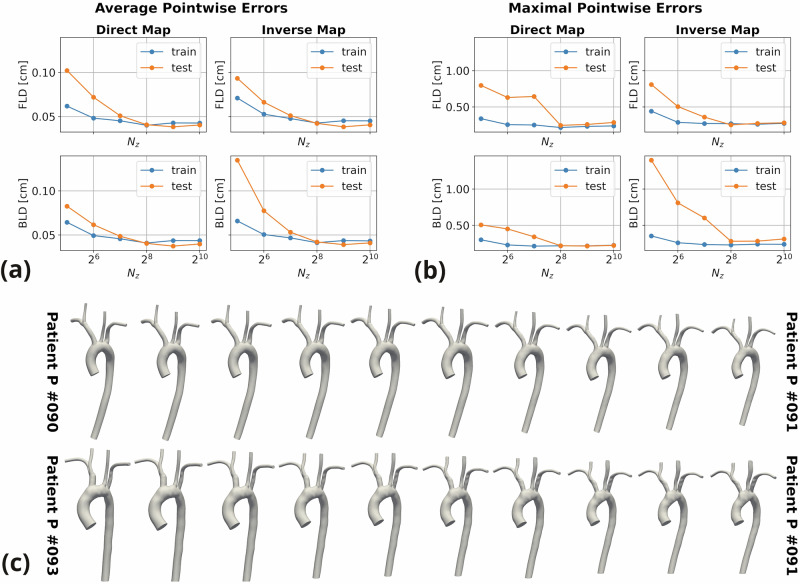
Deformable mapping results of the baseline AD-SVFD model. In particular, we report the average (**a**) and maximal (**b**) pointwise errors—quantified through the forward and backward local distances FLD and BLD, in cm—on training and testing datapoints, obtained for different shape code dimensions *N*_*z*_; in **c**, we show the geodesic paths between two source shapes (P#090 for training, P#093 for testing) and the reference shape (P#091), generated by numerical integration of the diffeomorphic flow equations by the forward Euler method at *K* = 10 intermediate steps.

Figure 3 reports the average (a) and maximal (b) FLD and BLD for both the direct and the inverse deformation, considering different values of *N*_*z*_. On the one hand, the results demonstrate that the latent space dimension should be taken sufficiently large in order to effectively condition the model weights towards accurate approximations of the diffeomorphic maps. On the other hand, we notice that model accuracy stalls for large values of *N*_*z*_, suggesting redundant information in the shape codes. Ultimately, we select *N*_*z*_ = 256 as the latent dimension, since it appears to optimally balance accuracy and efficiency. In terms of generalization power, we note that training and testing errors are comparable for *N*_*z*_ ≥ 256, thus indicating that no overfitting phenomenon occurs. Incidentally, we remark that no major discrepancy between the registration errors on the original and augmented testing geometries can be observed. For instance, only marginally lower maximal FLDs are obtained on the augmented geometries, both considering the direct and the inverse map (direct map errors: 0.2774 vs. 0.2822 cm; inverse map errors: 0.2562 vs. 0.2613 cm). In Fig. 3c, we appreciate how AD-SVFD smoothly and gradually warps the source shapes into the reference one, through the forward-in-time numerical integration of the learnable diffeomorphic flow equations (see Eq. (3)) by the explicit Euler method.

### Test 2: Data attachment measures

**Fig. 4:**
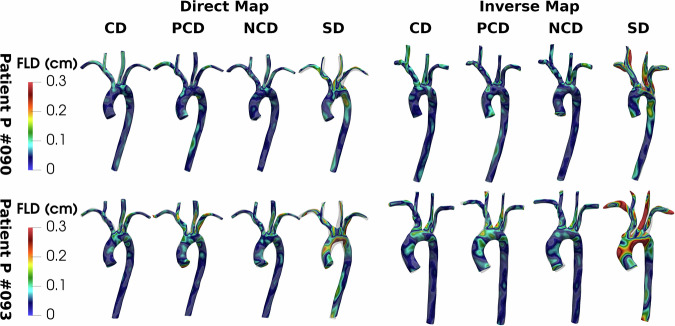
Registration results of AD-SVFD considering different data attachment measures. In particular, we show the direct and inverse mapping pointwise errors, obtained on a training (*P#090*) and a testing (*P#093*) datapoint, for four different data adherence metrics. The errors are quantified through the forward local distance (FLD), expressed in cm, namely the distance of each point in the mapped geometry from the closest one in the target. For reference, the inlet diameters are 1.31 cm for the template shape, 1.21 cm for *P#090*, and 1.32 cm for *P#093*. CD Chamfer distance, PCD point-to-surface Chamfer distance^48^, NCD Chamfer distance with normals penalization, SD debiased Sinkhorn divergence^49^.

We analyse the AD-SVFD model performances considering the different data attachment measures mentioned in the section “Introduction”. We refer to the “Methods” section for a detailed description of the different options and of their specifics. Table 1 reports the maximal pointwise errors on both training and testing datapoints. To quantitatively compare the results, we evaluate the maximal pointwise FLD and BLD, even when metrics different from the (unweighted) CD are used in the loss. Since this approach may introduce a bias in the analysis, we also provide a qualitative accuracy assessment through Fig. 4.

**Table 1:** Registration results of AD-SVFD considering different data attachment measures

	Train errors (in cm)				Test errors (in cm)			
	Direct		Inverse		Direct		Inverse	
Loss	FLD	BLD	FLD	BLD	FLD	BLD	FLD	BLD
${{\bf{{\mathcal{D}}}}}_{{\rm {CD}}}$	0.2162	0.2175	0.2686	**0.2297**	**0.2777**	**0.2253**	0.2562	**0.2642**
${{\bf{{\mathcal{D}}}}}_{{\rm {CD}}}^{{\rm {W}}}$	0.2412	0.2497	0.2869	0.2564	0.4088	0.2479	0.2952	0.4283
${{\bf{{\mathcal{D}}}}}_{{\rm {PCD}}}$	0.3195	**0.2165**	0.3225	0.2611	0.3138	0.2516	0.2749	0.3540
${{\bf{{\mathcal{D}}}}}_{\rm {{PCD}}}^{{\rm {W}}}$	0.2515	0.2510	0.2958	0.2579	0.3489	0.2439	0.3043	0.3686
${{\bf{{\mathcal{D}}}}}_{{\rm {NCD}}}$	**0.2033**	0.2166	**0.2628**	0.2396	0.2965	0.2260	**0.2497**	0.3090
${{\bf{{\mathcal{D}}}}}_{{\rm {SD}}}$	0.4887	0.3795	0.5355	0.3923	0.4519	0.4695	1.1094	0.4384
${{\bf{{\mathcal{D}}}}}_{{\rm {SD}}}^{{\rm {W}}}$	0.5866	0.3861	0.5155	0.3917	0.4156	0.4155	0.8275	0.4420

In particular, we report the maximal pointwise errors on training and testing datapoints, obtained for six different data adherence metrics. The errors are quantified through the forward and backward local distances (FLD and BLD), expressed in cm. The best value for each performance metric is shown in bold. For reference, the template shape inlet diameter is 1.31 cm, while the average inlet diameter in the dataset is 1.45 cm. CD: Chamfer distance; PCD: point-to-surface Chamfer distance^48^; NCD: Chamfer distance with normals penalization; SD: debiased Sinkhorn divergence^49^. Notation: the W superscript denotes the use of a weighted measure.

From both a quantitative and a qualitative standpoint, the best results are obtained considering the baseline model, which employs unweighted CD as a data attachment measure. Indeed, this model yields precise geometry reconstructions on both training and testing shapes, and it is associated with the lowest training time (equal to 7 h40 min) and with an average testing time of just $1\,\min 28\,{\rm{s}}$ per shape. Incorporating a penalization of the normals’ orientation in the loss (with *w*_n_ = 10^−2^) allows for marginal accuracy improvements, but at the cost of a much larger training time (equal to $16\,{\rm{h}}07\,\min$), due to the increased number of model evaluations. Incidentally, the larger memory requirements induced by the normals’ calculation prevent the use of L-BFGS at inference. To mitigate this issue, we replace NCD with unweighted CD at testing; this allows us to retain acceptable accuracy levels, even though worse than the training ones, at equivalent inference times. Neither leveraging the weights associated with the point clouds nor adopting PCD improves the mapping quality; in fact, both approaches are substantially outperformed by the baseline model. With a specific focus on PCD, from Fig. 4, we can observe marked discrepancies at the upper branches, which, in the case of patient *P#093* tend to squeeze into unrealistic flat morphologies. Lastly, we remark that the registration quality gets considerably worse when using debiased SD. Indeed, the deformed geometries take unlikely convoluted shapes, which become twisted and almost flat in the upper branches region. From a quantitative point of view, this translates into errors that roughly double the ones obtained with CD. Furthermore, compared to the baseline model, the heavier costs associated with the calculation of SD entail drastic increases in the durations of both training (from $7\,{\rm{h}}40\,\min$ to $27\,{\rm{h}}15\,\min$) and testing (from $1\,\min 28\,{\rm{s}}$ to $3\,\min 08\,{\rm{s}}$ per shape on average).

### Test 3: Comparison with state-of-the-art methods

To fairly assess the capabilities of AD-SVFD, we run a comparison test with five alternative shape registration models. Specifically, we evaluate the mapping quality for two different source shapes: *P#090* (training) and *P#278* (testing). We investigate the following models: coherent point drift (CPD)^40^, thin-plate spline (TPS) interpolation^54^ (see Supplementary Note 1 for details), LDDMM^9^, SDF4CHD^33^ and ResNet-LDDMM^28^. Furthermore, we also consider the SVFD model, namely the AD-SVFD model, lacking the auto-decoder structure. We note that all deformable registration models operate on anatomies that have been rigidly pre-aligned using the CPD method, whose registration errors therefore identify a baseline. A few aspects deserve attention.

- Except for SDF4CHD, all the approaches perform single shape-to-shape registrations, without leveraging any form of implicit geometry representation. Hence, for these models, there is no distinction between training and testing shapes.
- Using CPD, TPS and LDDMM, we can only estimate a one-directional map, warping the source shape into the reference one or vice versa. Therefore, the direct and inverse maps are retrieved by running two independent optimization processes. While this approach may improve registration accuracy, it comes at the cost of increased computational efforts and does not guarantee that the two maps compose to the identity.
- As reported in ref. ^28^, despite learning a diffeomorphism between two shapes, the ResNet-LDDMM model is solely optimized considering the source-to-template map result. To enhance inverse mapping quality, we introduce the *I-ResNet-LDDMM* model. Compared to the baseline, the latter solves a multi-objective optimization problem, including both direct and inverse mapping results within the loss. To provide a fair comparison with AD-SVFD, we employ CD as a data attachment measure, rely on the modified Euler scheme to integrate the diffeomorphic flow ODE backward in time, and equally weigh direct and inverse errors.

Regarding the models’ specifics, for ResNet-LDDMM and SDF4CHD, we employ the “optimal” model structures and hyperparameter sets, as identified in refs. ^28,33^, respectively. Furthermore, with SDF4CHD, we do not exploit the DeepSDF model^31^ to learn the SDF representation of an *Atlas* shape; instead, we use the pre-computed SDF of patient *P#091* to serve as a reference. For LDDMM, we rely on the *Deformetrica*^9^ software, and perform single shape-to-shape registration employing L-BFGS as optimization algorithm and the varifold distance as data attachment measure, with a Gaussian kernel of width 0.8. This last value, which leads to the optimization of roughly 1, 500 control points and momenta vectors, has been manually calibrated to balance efficiency and accuracy. For SVFD, we consider the optimal set of hyperparameters found during the calibration tests (see Supplementary Note 3).

**Fig. 5:**
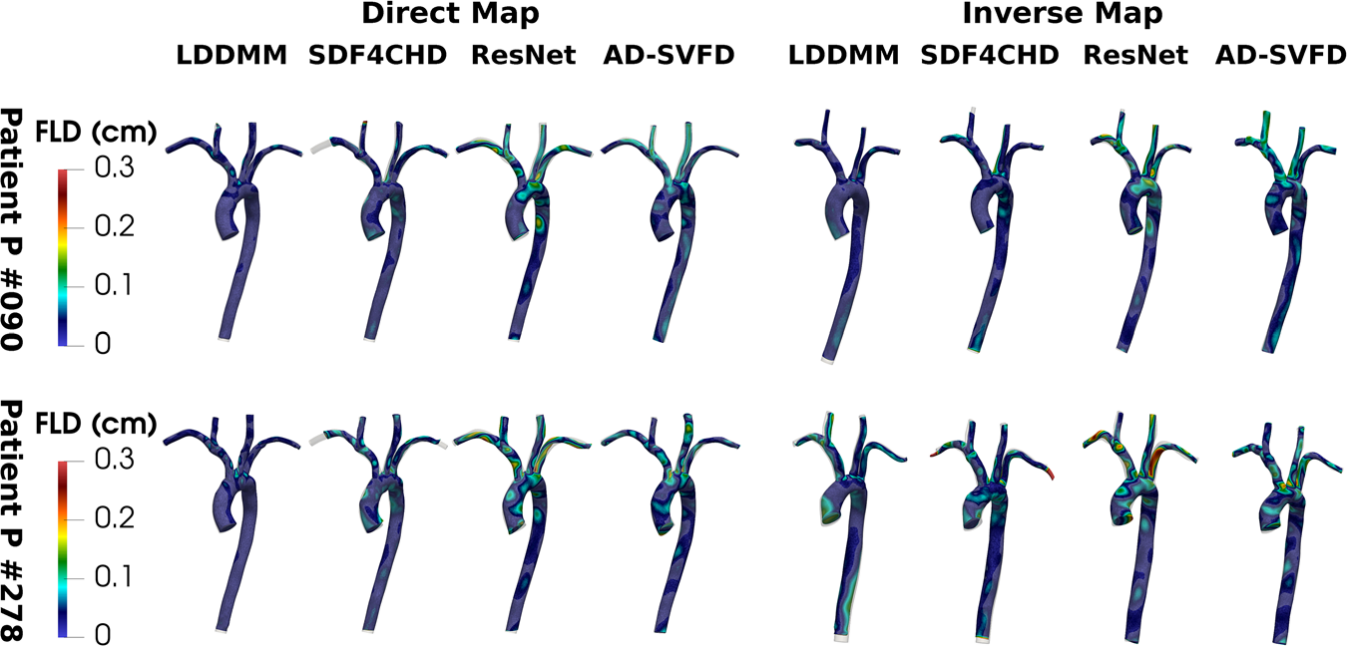
Registration results obtained with AD-SVFD and three alternative approaches. In particular, we show the direct and inverse mapping pointwise errors, obtained with LDDMM^9^, SDF4CHD^33^, ResNet-LDDMM^28^ and AD-SVFD on a training (*P#090*) and a testing (*P#278*) datapoint. The errors are quantified through the forward local distance (FLD), expressed in cm, namely the distance of each point in the mapped geometry from the closest one in the target. For reference, the inlet diameters are 1.31 cm for the template shape, 1.21 cm for *P#090*, and 1.52 cm for *P#278*.

Table 2 reports the maximal pointwise errors of the direct and inverse mappings obtained on the two considered source shapes, for the different registration models. Figure 5 displays the results for four of the models. In summary, we observe that SVFD and LDDMM outperform all the other approaches, while AD-SVFD trades a small accuracy deterioration for improved efficiency and generalization properties. Indeed, by direct comparison with SVFD, a registration error increase of 20% on *P#090* and 30% on *P#278* is counterbalanced by a drastic reduction of inference times—from roughly $12\,\min$ to $1\,\min 30\,{\rm{s}}$ —and by the capability of providing a unified framework to process an entire cohort of shapes. We note that LDDMM yields very precise approximations of the direct map, but its performances deteriorate and falls behind ones of AD-SVFD on the inverse map, particularly because of discrepancies at the inlet/outlet faces. A similar consideration holds for the SDF4CHD model, which is capable of producing anatomies that closely match the target ones, but that often feature artifacts and/or completely miss the final portion of the smallest branches. In contrast with the results reported in ref. ^28^, the residual neural network structure of ResNet-LDDMM does not allow it to outperform the canonical LDDMM method. Nonetheless, we acknowledge that fine-tuning the model hyperparameters to the present test case may sensibly improve the results. Additionally, we underline that the introduction of a penalty on the inverse mapping in ResNet-LDDMM determines minor but tangible improvements on all metrics.

**Table 2:** Comparison test of AD-SVFD with seven alternative registration methods

	Method	Multi shape	DL	Max. errors (in cm)			
				Direct		Inverse	
				FLD	BLD	FLD	BLD
P#090	CPD	✗	✗	1.4321	2.0983	1.3985	2.9714
	TPS	✗	✗	0.2918	0.2615	0.4305	0.3674
	LDDMM	✗	✗	0.1813	**0.1227**	0.2625	0.3332
	ResNet	✗	*✓*	0.2470	0.2806	0.4393	0.6520
	I-ResNet	✗	*✓*	0.1948	0.2269	0.2139	0.2466
	SVFD	✗	*✓*	**0.1339**	0.1479	**0.1674**	**0.1659**
	SDF4CHD	*✓*	*✓*	0.3861	1.7831	0.6250	0.4207
	AD-SVFD	*✓*	*✓*	0.1693	0.1923	0.2071	0.1719
P#278	CPD	✗	✗	1.5265	1.1772	1.3802	2.0471
	TPS	✗	✗	0.5092	0.3521	0.7974	0.7104
	LDDMM	✗	✗	**0.1281**	0.1681	0.5160	0.5441
	ResNet	✗	*✓*	0.3085	0.2720	0.3546	0.3594
	I-ResNet	✗	*✓*	0.2805	0.2498	0.2986	0.3367
	SVFD	✗	*✓*	0.1343	**0.1614**	**0.2248**	**0.2259**
	SDF4CHD	*✓*	*✓*	0.2598	1.2918	1.0277	0.2754
	AD-SVFD	*✓*	*✓*	0.2166	0.1807	0.2817	0.2933

In particular, we report the maximal pointwise errors on patients *P#090* and *P#278*, obtained considering CPD^40^, TPS^54^, LDDMM^9^, ResNet-LDDMM ^28^ (optionally endowed with a penalty of the inverse deformation, I-ResNet-LDDMM), SVFD (i.e., our model without auto-decoder structure), SDF4CHD^33^, and AD-SVFD. The errors are quantified through the forward and backward local distances (FLD and BLD), expressed in cm. The column *Multi Shape* identifies methods that allow for the simultaneous registration of multiple shapes; the column *DL* identifies deep learning models. The best results for single-shape approaches are shown in bold; the best results for multi-shape approaches are underlined. For reference, the inlet diameters are: 1.31 cm for *P#091* (template); 1.22 cm for *P#090*; 1.52 cm for *P#278*.

### Test 4: Robustness assessment

To save computational resources, all numerical experiments described so far were conducted in a “fixed” scenario, namely, for the same random initialization of the trainable parameters and reserving the same patients (*P#093*, *P#278*) for testing. This way of proceeding prevents a thorough assessment of robustness, which is of paramount importance in DL applications. To this aim, we perform a 10-fold cross-validation, designed with respect to the “original” geometries in the dataset. This means that, if the anatomy of a given patient is reserved for testing, then all the augmented versions of such anatomy are not considered for training. For each fold, we run three independent training processes, considering different random seeds. For this test, both training and testing errors are computed solely accounting for original anatomies.

Table 3 reports the obtained results, in terms of training and testing FLD and BLD, for both the direct and the inverse mapping. We observe that all models yield precise approximations of the diffeomorphic maps on the training datapoints, with maximal pointwise errors that always lie below the 0.30 cm threshold. However, markedly larger errors are produced at testing, in particular for folds #1, #2, #8, and #10. This phenomenon can be explained by considering that these folds, respectively, reserve for testing patients *P#275*, *P#207*, *P#188*, *P#205*, whose geometries present features that are uniquely represented within the dataset. For instance (see Fig. 2): patient *P#207* is characterized by the only anatomy whose RSA bends towards (and not away from) RCCA; patient *P#205* is the only one whose horizontal LSA chunk could not be segmented. Hence, the drop in precision can be ascribed to data paucity.

**Table 3:** Cross-validation procedure results

		Train errors (in cm)				Test errors (in cm)			
		Direct		Inverse		Direct		Inverse	
Fold #	Test P#	FLD	BLD	FLD	BLD	FLD	BLD	FLD	BLD
1	090, 275	0.2439	0.2367	0.2775	0.2401	0.5000	0.3153	0.3708	0.5747
2	091, 207	0.2406	0.2174	0.2692	0.2397	0.6429	0.2690	0.2512	0.6902
3	139, 143	0.2232	0.2171	0.2704	0.2259	0.3091	0.2687	0.2710	0.2638
4	093, 187	0.1984	0.2125	0.2641	0.2252	0.3485	0.2458	0.2400	0.4382
5	272, 277	0.2390	0.2418	0.2702	0.2312	0.3119	0.2281	0.2856	0.3770
6	092, 201	0.2351	0.2210	0.2748	0.2291	0.2236	0.2350	0.2429	0.2745
7	144, 278	0.2307	0.2166	0.2675	0.2360	0.3484	0.2328	0.2950	0.3608
8	094, 188	0.2252	0.2228	0.2639	0.2267	0.6567	0.3454	0.3602	0.4967
9	142, 145	0.2120	0.2046	0.2525	0.2225	0.2901	0.2867	0.3414	0.2676
10	141, 205	0.2205	0.2170	0.2628	0.2260	0.5114	0.4143	0.3124	0.3639
Avg.		0.2269	0.2208	0.2673	0.2302	0.4143	0.2841	0.2971	0.4107
Std.		0.0134	0.0104	0.0067	0.0060	0.1448	0.0564	0.0455	0.1340

In particular, we report the maximal pointwise errors on training and testing datapoints, solely considering original anatomies, obtained with the AD-SVFD model for the 10 different folds during cross-validation. The errors are quantified through the forward and backward local distances (FLD and BLD), expressed in cm. The reported results are averages that stem from three independent training procedures, conducted by setting different random seeds. Underlined IDs in *Test P#* column denote patients that have been considered for comparison tests with alternative shape registration methods; we refer to Supplementary Note 4 for details. For reference, the template shape inlet diameter is 1.31 cm, while the average inlet diameter in the dataset is 1.45 cm.

To further evaluate the robustness of AD-SVFD, we exploit the cross-validation results to compare its performance on 10 testing patients (one per fold, see underlined values in Table 3) to those of LDDMM, I-ResNet-LDDMM and SVFD. We refer to Supplementary Note 4 for the details. From a general standpoint, the obtained results highlight the good generalization capability of AD-SVFD. Although its average accuracy is slightly lower than that of LDDMM (+5% and +16% on the direct and inverse map errors) and I-ResNet-LDDMM (+22% and +5% on the direct and inverse map errors), this drawback is largely offset by a substantial reduction of the inference time (−85% compared to LDDMM, from 10 to 1.5 min, and −81% compared to I-ResNet-LDDMM, from 8 to 1.5 min). Moreover, we note that the SVFD model outperforms the two competing single-shape registration approaches, achieving 56% and 33% lower direct and inverse map errors compared to LDDMM, and 39% and 49% lower direct and inverse map errors compared to I-ResNet-LDDMM. Additionally, it provides accurate predictions even for the “critical” patients, *P#205* and *P#207*. These findings confirm that the use of latent shape codes, albeit enabling faster inference and generative applications, inevitably entails accuracy losses and performance pitfalls, largely attributable to data scarcity.

### Test 5: Latent space analysis and generative modelling

The use of low-dimensional latent codes, belonging to the learnable space ${\mathcal{Z}}$, makes AD-SVFD suited for generative modelling. Indeed, once the model is trained, new anatomies can be generated by sampling shape code instances from ${\mathcal{Z}}$ and applying the corresponding inverse maps to the template geometry. We highlight that the robustness of the generative process is intimately related to the latent space regularity. For this reason, we include a penalization of the shape code entries in the loss, weighted by the positive constant *ω*_*z*_ (see Eq. (11)).

**Fig. 6:**
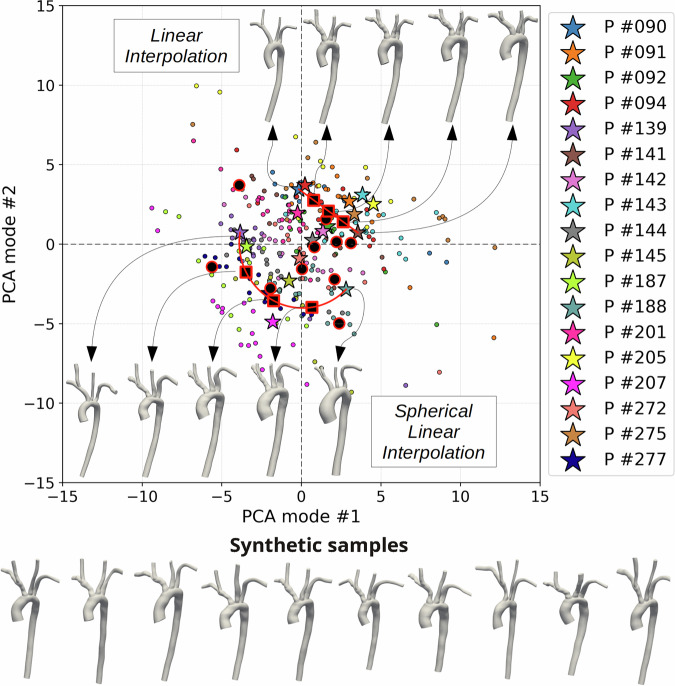
Latent space learned by the AD-SVFD model and generated synthetic anatomies. We show the projection of the shape codes onto the two-dimensional subspace obtained through PCA on the whole set of training codes. We report the latent codes of the original patients (stars), and, for each of those, the latent codes of 20 associated augmented geometries (circles). Furthermore, we report 10 entries sampled from ${\mathcal{N}}({\boldsymbol{0}},{\Sigma }_{z})$, where *Σ*_*z*_ is an unbiased estimate of the covariance matrix computed from the training codes (red& black circles); their synthetic counterparts in the physical space are displayed at the bottom of the image. Finally, we report the results obtained through interpolation in the latent space. On the top margin, we show 3 entries generated via linear interpolation between *P#090* and *P#141*; at the bottom margin, we show 3 entries generated via spherical linear interpolation between *P#139* and *P#188*. The black arrows map shape code instances to their physical counterparts.

Figure 6 reports a sketch of the latent space learned by the AD-SVFD model. We show the projections of the shape codes associated with the training source anatomies onto a two-dimensional subspace, obtained through principal component analysis (PCA). Furthermore, we display 10 entries that are randomly sampled from ${\mathcal{N}}({\boldsymbol{0}},{\Sigma }_{z})$ —where *Σ*_*z*_ is an unbiased estimate of the covariance matrix computed from the training shape codes (red and black circles)—and we report their physical counterparts. Finally, we illustrate the results stemming from interpolation in the latent space; in particular, we display 3 synthetic anatomies generated via linear interpolation between *P#090* and *P#141*, and 3 synthetic anatomies generated via spherical linear interpolation (SLERP)^57,58^ between *P#139* and *P#188* (red&black squares). Based on the obtained results, we can claim that the learned latent space exhibits satisfactory smoothness and regularity properties, at least from a qualitative standpoint. Indeed, with reference to Fig. 2, geometries that are similar in the physical space are characterized by close embeddings in ${\mathcal{Z}}$ (e.g., *P#090* and *P#094* or *P#092* and *P#142*). On the contrary, shape codes associated with anatomies that feature unique traits within the dataset lie in more isolated areas at the boundary of ${\mathcal{Z}}$ (e.g., *P#207*). The interpolation results provide additional evidence on the regularity of ${\mathcal{Z}}$. Indeed, both in the case of linear and spherical linear interpolation, we observe a smooth variation of the anatomies without major artifacts: branch count is preserved, lumen area stays positive, and centerline curves evolve smoothly. From a quantitative perspective, we noted that the Chamfer distance from the target patient (i.e., *P#141* and *P#188* in the reported experiments) regularly varies over the path.

Additional numerical tests show that noisy versions of the shape codes of the original patients, obtained by adding white Gaussian noise, result in synthetic anatomies whose discrepancy with respect to the target is small and proportional to the noise level. For instance, in the case of patient *P#094*, shape codes with different signal-to-noise ratios (SNR) yield anatomies characterized by the following FCD and BCD values: 0.2089 and 0.1993 cm for SNR = 1%, 0.2118 and 0.2022 cm for SNR = 2%, 0.2212 and 0.2117 cm for SNR = 5%, and 0.2389 and 0.2283 cm for SNR = 10%. For reference, the FCD and BCD between *P#094* and *P#090*, whose codes differ by 16% in Euclidean norm, are 0.1921 and 0.2544 cm, respectively. Also, the FCD and BCD between *P#094* and its mapped version through AD-SVFD, namely what stems from setting SNR = 0%, are 0.0367 and 0.0354 cm, respectively. These results provide empirical evidence on the stability of the latent space.

## Discussion

We introduced AD-SVFD, a deep learning model for the non-rigid registration and synthetic generation of three–dimensional surfaces, tailored to vascular anatomies and, in particular, to healthy aortas.

Analogously to refs. ^27,28^, the AD-SVFD model performs 3D point cloud registration, leveraging shape representations in the form of weighted point clouds, whose weights are proportional to the nearest neighbours distance (see Eq. (1)). In this regard, AD-SVFD differs from deformable registration models based on continuous signed distance functions (SDFs), such as the ones presented in refs. ^32,33^. As empirically demonstrated in *Test 3* through a comparison of the performances of AD-SVFD and SDF4CHD, this approach enables more precise reconstructions, at least for vascular anatomies. Indeed, as shown in Fig. 5, AD-SVFD clearly outperforms SDF4CHD^33^, whose deformed anatomies either omit or severely distort most of the smallest branches. This phenomenon can plausibly be attributed to the use of SDFs, whose resolution must remain limited for computational efficiency reasons, thereby hindering the accurate capture of the finest details. Notably, the outcomes of *Test 3* also reveal two additional key aspects. On the one hand, AD-SVFD demonstrates superior performance, in both accuracy and efficiency, compared to DL-based single-shape 3D point cloud registration methods such as ResNet-LDDMM^28^. On the other hand, traditional approaches like LDDMM^9^, not rooted in DL techniques, exhibit comparable accuracy metrics but are significantly more computationally demanding at inference. In fact, the most accurate single-shape registration approach in the context of interest is SVFD, namely, our model lacking the auto-decoder structure. This result suggests that the adoption of shape embeddings allows for faster inference and generative use cases, but also implies precision losses.

Dealing with point clouds in the absence of ground-truth point-to-point correspondences required the consideration of alternative data attachment measures, both to construct an effective loss function and to design informative error indicators. This aspect was analysed in *Test 2*, where multiple data adherence metrics were investigated. Although representing the baseline alternative, the canonical (i.e., unweighted) Chamfer Distance outperforms all other options, delivering the most precise geometry reconstructions at the lowest computational costs and memory requirements. In the test case at hand, neither incorporating the normals’ orientation nor exploiting the point cloud weights resulted in improved precision, while instead inducing moderate to substantial increases in complexity. Notably, the performance achieved using the debiased Sinkhorn divergence in the loss proved unsatisfactory in terms of both accuracy and efficiency, as also observed in ref. ^28^ on non-elementary geometries.

As in the model proposed in ref. ^27^, AD-SVFD expresses diffeomorphic maps through the *stationary vector field* parametrization. Specifically, the ambient space deformation is defined as the solution at unit time of a system of ODEs, whose learnable right-hand side does not explicitly depend on time (see Eq. (3)). In particular, the right-hand side is modeled by a fully-connected and *Leaky-ReLU* activated ANN, so as to ensure well-posedness. By numerically integrating the diffeomorphic flow equations over time, it becomes possible to reconstruct the geodesic paths connecting source anatomies to the template. As illustrated in Fig. 3 (bottom), this procedure gives rise to a collection of synthetic shapes, exhibiting a smooth and gradual transition from the source characteristics to the reference ones.

Extracting the intermediate stages of numerical integration is not the only means of generating artificial geometries with AD-SVFD. Indeed, a crucial feature of the proposed model, distinguishing it, for instance, from ResNet-LDDMM^28^, is the internalization of latent embeddings for the source anatomies. Similar to the models presented in refs. ^32,33^, this is accomplished by introducing low-dimensional shape codes, which serve as trainable input variables within an auto-decoder architecture. Consequently, the available geometries are mapped onto a low-dimensional latent space, where convenient random sampling routines can be implemented for generative purposes. Further details regarding the definition and treatment of shape codes are provided in the “Methods” section. In *Test 1*, it was demonstrated that the dimension of the latent space, denoted as *N*_*z*_, should be carefully calibrated to optimally balance precision and performance. As illustrated in Fig. 3 (top), accuracy is significantly compromised when excessively small shape codes are employed, while it plateaus for large values of *N*_*z*_, where model complexity and memory demands become prohibitive instead.

In addition to controlling the latent space dimension, monitoring its regularity is of paramount importance to ensure the robustness and reliability of downstream generative AI applications. To this end, a suitable penalization term was included in the loss function (see Eq. (4)), with its weight *w*_*z*_ = 10^−3^ carefully fine-tuned. As discussed in *Test 5* and illustrated in Fig. 6, this strategy ultimately enables the construction of a smooth latent space that can be robustly queried to generate customizable, realistic, synthetic anatomies. It is worth noting that a well-established and widely adopted technique to enforce latent space regularity consists of variational training. Accordingly, several exploratory experiments were conducted in this direction, updating both the model structure and the loss function to implement a variational auto-decoder formulation for AD-SVFD^59^. However, no substantial improvements in regularity or robustness were observed, while approximation quality was markedly degraded.

Despite exhibiting highly promising results, the current work nonetheless presents certain limitations. First and foremost, as with many DL-based models, data availability imposes non-negligible performance constraints, which could only be partially mitigated through data augmentation. This issue becomes particularly evident from the cross-validation results reported in *Test 4*. Specifically, AD-SVFD accuracy on unseen anatomies declines for folds containing testing shapes featuring unique traits within the dataset, such as *P#205* and *P#207* (see Fig. 2). It is noteworthy that additional aortic anatomies from the *Vascular Model Repository* were also considered during preliminary stages. However, they were subsequently discarded due to incompatibility with the adopted data augmentation pipeline, which produced undesired non-physiological artifacts. Incidentally, although the conducted tests were limited to healthy aortas, it is important to emphasize that the proposed registration approach is general and can be seamlessly extended to a wide range of challenging applications. Secondly, to reduce computational effort, most hyperparameters were fine-tuned within a simplified single shape-to-shape registration setting, as detailed in Supplementary Note 3. In practice, only the hyperparameters associated with the shape codes (*N*_*z*_, *w*_*z*_, and *λ*_*z*_) were calibrated using the full AD-SVFD model. Consequently, at least marginal performance improvements may be achievable through hyperparameter configurations specifically tailored to a multi-shape context. Lastly, the present analysis focused on the size and regularity of the latent space, but it did not address its interpretability. Investigating this aspect may enhance the generative pipeline and will therefore be the subject of future studies.

In conclusion, AD-SVFD can serve as a valuable tool for engineering applications involving physical problems in complex geometries. The proposed approach introduces potentially distinctive elements for facilitating geometry manipulation, notably by simultaneously providing compact and portable representations and by learning accurate, smooth, invertible, and topology-preserving mappings to a pre-defined reference. Notwithstanding improvements of its generalization capabilities, AD-SVFD is envisioned as a pre-trained module within physics-aware machine learning frameworks, enabling the incorporation of realistic geometrical variability in the simulation of complex physical processes^60–64^.

## Methods

We provide a more detailed analysis of the AD-SVFD model, specifically focusing on the shape codes, the ANN architecture, the numerical integration of the flow equations, the data attachment measures and the optimization procedure.

### Latent shape codes

AD-SVFD provides a unified framework for the simultaneous registration of the source anatomies to a pre-defined template, leveraging implicit neural representations. Indeed, every source shape ${{\mathcal{S}}}_{i}$ is associated with a shape code ${{\boldsymbol{z}}}_{i}\in {{\mathbb{R}}}^{{N}_{z}}$, so that the diffeomorphism ${\overrightarrow{\varphi }}_{i}$ mapping ${{\mathcal{S}}}_{i}$ to ${\mathcal{T}}$ configure as the specialized version of a “generic” diffeomorphism $\vec{\varphi }$, i.e. ${\vec{\varphi }}_{i}({\boldsymbol{x}}):= \vec{\varphi }({\boldsymbol{x}},{{\boldsymbol{z}}}_{i})$, with ${\boldsymbol{x}}\in {{\mathbb{R}}}^{3}$.

Instead of directly providing the latent codes in input to the model, we borrow from ref. ^33^ the use of a position-aware shape encoding strategy^65^. Given a shape code ***z***_*i*_, we define the associated shape code grid ${{\boldsymbol{Z}}}_{i}\in {{\mathbb{R}}}^{{g}_{z}\times {g}_{z}\times {g}_{z}\times ({N}_{z}/{g}_{z}^{3})}$ as ${{\boldsymbol{Z}}}_{i}={{\mathcal{R}}}_{z}({{\boldsymbol{z}}}_{i})$, where ${{\mathcal{R}}}_{z}:{{\mathbb{R}}}^{{N}_{z}}\to {{\mathbb{R}}}^{{g}_{z}\times {g}_{z}\times {g}_{z}\times ({N}_{z}/{g}_{z}^{3})}$ is a suitable reshaping function. Here, we suppose that the shape code dimension *N*_*z*_ is a multiple of ${g}_{z}^{3}$; in this work, we always select *g*_*z*_ = 2. Then, for a given point ***x*** ∈ Ω, the position-aware shape code ${\bar{{\boldsymbol{z}}}}_{i}({\boldsymbol{x}})\in {{\mathbb{R}}}^{{N}_{z}/{g}_{z}^{3}}$, associated with the source shape ${{\mathcal{S}}}_{i}$, is obtained by evaluating the trilinear interpolation of ***Z***_*i*_ at ***x***, i.e. ${\bar{{\boldsymbol{z}}}}_{i}({\boldsymbol{x}}):= {\rm{Lerp}}({\boldsymbol{x}},{{\boldsymbol{Z}}}_{i})$, being Lerp(⋅ , ⋅) the trilinear interpolation function. This approach comes with two major advantages. On the one hand, the positional awareness of the latent codes helps the model in better differentiating the deformation flow field, depending on the location within the domain. On the other hand, even if the total number of trainable parameters is unchanged, only $({N}_{z}/{g}_{z}^{3})$-dimensional vectors are provided as input to the ANN. Hence, model complexity is (slightly) reduced compared to the *naive* approach, ideally at no loss in representation power.

### Artificial neural network architecture

To learn the diffeomorphisms between the source anatomies and the template, we exploit the Neural ODE approach^34^, employing an ANN to approximate the right-hand side of Eq. (3). More specifically, we consider a DL-based structure comprising three modules:

- Feature augmentation network (FA-NN): The first part of the model performs a data-driven feature augmentation of the input locations. It consists of a fully connected ANN that takes as input a spatial location ***x*** ∈ *Ω* and the associated position-aware shape code ${\bar{{\boldsymbol{z}}}}_{i}({\boldsymbol{x}})$ and yields a set of latent features ${{\boldsymbol{x}}}_{i,{\rm {FA}}}\in {{\mathbb{R}}}^{{N}_{{\rm {FA}}}}$ as output. Since FA-NN solely acts as a feature augmentation compartment, we do not want it to weigh down model complexity. So, we consider shallow networks with few neurons per layer. We highlight that the learned features are anatomy-dependent, thanks to the conditioning effect of the shape code on the model weights. We can summarize the FA-NN action via the function ${{\mathcal{F}}}_{{\rm {FA}}}:{{\mathbb{R}}}^{3}\times {{\mathbb{R}}}^{{N}_{z}/{g}_{z}^{3}}\to {{\mathbb{R}}}^{{N}_{{\rm {FA}}}}$, such that ${{\boldsymbol{x}}}_{i,{\rm {FA}}}={{\mathcal{F}}}_{{\rm {FA}}}({\boldsymbol{x}},{\bar{{\boldsymbol{z}}}}_{i}({\boldsymbol{x}});{\mathbf{\Theta }})$.
- Fourier Positional Encoder (FPE): In the second module, the learned latent features ***x***_*i*,FA_ undergo a further augmentation step via a deterministic Fourier positional encoding^66^, adopting a base-2 logarithmic sampling strategy in the frequency domain. This step is crucial for mitigating the spectral bias of ANNs^67^. We can summarize the FPE action via the function ${{\mathcal{F}}}_{{\rm {FPE}}}:{{\mathbb{R}}}^{{N}_{{\rm {FA}}}}\to {{\mathbb{R}}}^{{N}_{{\rm {NPE}}}}$, such that ${{\boldsymbol{x}}}_{i,{\rm {FPE}}}={{\mathcal{F}}}_{{\rm {FPE}}}({{\boldsymbol{x}}}_{i,{\rm {FA}}})$, where *N*_NPE_ ≔ (2*N*_e_ + 1)*N*_FA_.
- Diffeomorphic flow network (DF-NN): The last chunk of the ANN model is responsible for the approximation of the stationary velocity field at the spatial location ***x*** — the right-hand side of Eq.(3)—given the augmented features ***x***_*i*,FPE_ and the position-aware shape code ${\bar{{\boldsymbol{z}}}}_{i}({\boldsymbol{x}})$. As for FA-NN, we use a fully connected ANN. However, since DF-NN is the core part of the model, we consider deeper architectures with a larger number of neurons in each layer. We underline that the output of DF-NN depends on the source anatomy, thanks to the external conditioning effect of the shape codes. We can summarize the DF-NN action via the function ${{\mathcal{F}}}_{{\rm {DF}}}:{{\mathbb{R}}}^{{N}_{{\rm {FPE}}}}\times {{\mathbb{R}}}^{{N}_{z}/{g}_{z}^{3}}\to {{\mathbb{R}}}^{3}$, such that ${{\boldsymbol{v}}}_{i}={{\mathcal{F}}}_{{\rm {DF}}}({{\boldsymbol{x}}}_{i,{\rm {FPE}}},{\bar{{\boldsymbol{z}}}}_{i}({\boldsymbol{x}});{\mathbf{\Theta }})$.

Ultimately, we can express the action of the entire ANN model by the function ${\mathcal{F}}:{{\mathbb{R}}}^{3}\times {{\mathbb{R}}}^{{N}_{z}/{g}_{z}^{3}}\to {{\mathbb{R}}}^{3}$, defined as ${\mathcal{F}}:= {{\mathcal{F}}}_{{\rm {FA}}}\circ {{\mathcal{F}}}_{{\rm {FPE}}}\circ {{\mathcal{F}}}_{{\rm {DF}}}$. As resulting from the hyperparameter tuning procedure (see Supplementary Note 3), we consider: (i) *Leaky-ReLU* activation functions, with negative slope of 0.2, for both FA-NN and DF-NN; (ii) FA-NN with 3 layers of dimension 64; (iii) DF-NN with 5 layers of dimension 256; (iv) *N*_e_ = 3 for the FPE. Neglecting the latent codes’ contributions, the ANN model counts ~278k trainable parameters. To ease the notation, in the following, we omit the explicit spatial dependency of the shape codes.

### Numerical integration of the flow equations

For the numerical integration of the diffeomorphic flow equations (see Eq. (3)), we rely on first-order methods. Specifically, for the forward-in-time direct mapping, we employ the explicit forward Euler method. Let ${{\boldsymbol{x}}}_{i,j}^{s,(0)}={{\boldsymbol{x}}}_{i,j}^{s}\in \Omega$ be a point of the source point cloud ${{\mathcal{S}}}_{i}$, where the superscript (0) denotes the initial iteration count. Also, let $K\in {\mathbb{N}}$ be the number of time steps; in all tests, we set *K* = 10. Then, for *k* < *K*, the time marching scheme proceeds as follows:

5 $${{\boldsymbol{x}}}_{i,j}^{s,(k+1)}={{\boldsymbol{x}}}_{i,j}^{s,(k)}+\frac{1}{K}{\mathcal{F}}\left({{\boldsymbol{x}}}_{i,j}^{s,(k)},{\bar{{\boldsymbol{z}}}}_{i};{\mathbf{\Theta }}\right)={{\boldsymbol{x}}}_{i,j}^{s,(k)}+\frac{1}{K}{{\boldsymbol{v}}}_{i,j}^{s,(k)}\,.$$

The point corresponding to ${{\boldsymbol{x}}}_{i,j}^{s}$ in the template space is then the result of Eq. (5) at *k* = *K*−1, i.e ${{\boldsymbol{x}}}_{i,j}^{{\rm {s}},(K)}$.

To compute the inverse map, which deforms the ambient space so as to overlap the template anatomy with the source, we integrate the flow equations backward in time, given a final condition. In particular, we want the discrete inverse map to be the “true” inverse of the discrete direct map, defined in Eq. (5). So, let ${{\boldsymbol{x}}}_{i,j}^{{\rm {t}},(K)}={{\boldsymbol{x}}}_{j}^{{\rm {t}}}\in \Omega$ be a point of the template point cloud ${\mathcal{T}}$. The time marching scheme at step *k* > 0 proceeds as follows:

6 $${{\boldsymbol{x}}}_{i,j}^{{\rm {t}},(k-1)}={{\boldsymbol{x}}}_{i,j}^{{\rm {t}},(k)}-\frac{1}{K}{\mathcal{F}}\left({{\boldsymbol{x}}}_{i,j}^{{\rm {t}},(k-1)},{\bar{{\boldsymbol{z}}}}_{i};{\mathbf{\Theta }}\right)\,.$$

Even though Eq. (6) allows to invert Eq. (5) exactly, its use may be difficult in practice, being an implicit scheme. Indeed, the nonlinearity of ${\mathcal{F}}$ entails the use of ad hoc numerical techniques, such as Newton iterations, to compute a solution. Despite the Jacobian of ${\mathcal{F}}$ can be efficiently computed by automatic differentiation, the whole procedure is likely to slow down both the forward and the backward pass. For this reason, we rely on a first-order explicit approximation of Eq. (6)—known as the modified Euler scheme—that writes as follows:

7 $${{\boldsymbol{x}}}_{i,j}^{{\rm {t}},(k-1)}={{\boldsymbol{x}}}_{i,j}^{{\rm {t}},(k)}-\frac{1}{K}{\mathcal{F}}\left({{\boldsymbol{x}}}_{i,j}^{{\rm {t}},(k)}-\frac{1}{K}{\mathcal{F}}\left({{\boldsymbol{x}}}_{i,j}^{{\rm {t}},(k)},{\bar{{\boldsymbol{z}}}}_{i};{\mathbf{\Theta }}\right),{\bar{{\boldsymbol{z}}}}_{i};{\mathbf{\Theta }}\right)={{\boldsymbol{x}}}_{i,j}^{{\rm {t}},(k)}-\frac{1}{K}{{\boldsymbol{v}}}_{i,j}^{{\rm {t}},(k)}\,.$$

The point corresponding to ${{\boldsymbol{x}}}_{j}^{{\rm {t}}}$ in the source space is then the result of Eq. (7) at *k* = 1, i.e ${{\boldsymbol{x}}}_{i,j}^{{\rm {t}},(0)}$.

### Data attachment measures

As reported in Eq. (1), we represent three-dimensional surfaces as (weighted) point clouds, and we assume not to know exact point-to-point correspondences. Therefore, suitable data attachment measures to quantify the discrepancy between point clouds have to be considered. The simplest alternative is offered by the Chamfer Distance (CD) ${{\mathcal{D}}}_{{\rm {CD}}}:{{\mathbb{R}}}^{M\times 3}\times {{\mathbb{R}}}^{M^{\prime} \times 3}\to {{\mathbb{R}}}^{+}$, which is defined as

8 $${{\mathcal{D}}}_{{\rm {CD}}}(Y,{Y}^{{\prime} }):= \frac{1}{M}\mathop{\sum }\limits_{i=1}^{M}\mathop{\min }\limits_{{{\boldsymbol{c}}}^{{\prime} }\in {Y}^{{\prime} }}| | {Y}_{i}-{{\boldsymbol{c}}}^{{\prime} }| {| }_{2}^{2}\,+\,\frac{1}{M^{\prime} }\mathop{\sum }\limits_{{i}^{{\prime} }=1}^{M^{\prime} }\mathop{\min }\limits_{{\boldsymbol{c}}\in Y}| | {\boldsymbol{c}}-{Y}_{{i}^{{\prime} }}^{{\prime} }| {| }_{2}^{2}\,.$$

In particular, the CD comprises the sum of two terms: the forward CD (FCD), which compares the points in *Y* with the closest ones in ${Y}^{{\prime} }$, and the backward CD (BCD), which compares the points ${Y}^{{\prime} }$ with the closest ones in *Y*. Considering both components is crucial to obtain a meaningful goodness-of-fit measure. CD has proven to be an effective metric for diffeomorphic registration, particularly in the computational anatomy framework, as shown, e.g. in ref. ^28^. However, in ref. ^47^ it has been demonstrated that using CD is also likely to yield low-quality gradients. To mitigate this issue, we consider the earth mover’s distance (EMD) ${{\mathcal{D}}}_{{\rm {EMD}}}:{{\mathbb{R}}}^{M\times 3}\times {{\mathbb{R}}}^{M^{\prime} \times 3}\to {{\mathbb{R}}}^{+}$^68,69^:

$${{\mathcal{L}}}_{EMD}(Y,{Y}^{{\prime} })=\mathop{\min }\limits_{\xi \in {\mathcal{M}}(Y,{Y}^{{\prime} })}\sum _{{\boldsymbol{y}}\in Y}| | {\boldsymbol{y}}-\xi ({\boldsymbol{y}})| {| }_{2}^{2}\,,$$

where ${\mathcal{M}}(Y,{Y}^{{\prime} })$ denotes the set of 1-to-1 (bipartite) mappings from *Y* to *Y*${}^{{\prime} }$. In a nutshell, EMD is a Wasserstein distance that seeks the optimal transport plan that orders the points in ${Y}^{{\prime} }$ to match the ones in *Y*. In practice, we approximate EMD with the debiased Sinkhorn divergence (SD) ${{\mathcal{D}}}_{{\rm {SD}}}$^49^. The latter is the solution to an optimal transport problem with entropic constraints, and it can be estimated using the iterative Sinkhorn’s algorithm^70^. We refer the reader to ref. ^47^ for the precise definition of ${{\mathcal{D}}}_{{\rm {SD}}}$; further details and a state-of-the-art literature review on diffeomorphic registration using SD can be found in ref. ^71^. In all numerical tests conducted using SD, we consider a quadratic ground cost point function, a temperature scalar *ε* = 10^−4^, and a linear *ε*-scaling with factor 0.9. This combination of hyperparameters should be sensible for input measures that lie in the unit cube, providing a good trade-off between accuracy and efficiency^72^.

According to Feydy et al.^47^, SD is a good overlapping metric only if the points are roughly equispaced. However, SD can be effectively extended to unevenly distributed point clouds if the latter are weighted, i.e., if each point is associated with a quantity proportional to its distance from the closest neighbours. In fact, such weights appear in the entropic regularization term and in the entropic constraints of the associated optimal transport problem, awarding more “importance” to the most isolated points in the cloud. As reported in the “Introduction” section, in this work, we extract the cloud points as the cell centers of available surface triangulations and we compute the weights as the corresponding cell areas, normalized to add up to one. In the following, we denote by ${{\mathcal{D}}}_{{\rm {SD}}}$ the standard SD, where all the weights are assumed to be equal, and by ${{\mathcal{D}}}_{{\rm {SD}}}^{{\rm {W}}}$ the weighted SD. A similar reasoning can also be extended to the CD, even if the latter is not related to any optimal transport problem^73^. In this work, we define a weighted CD ${{\mathcal{D}}}_{{\rm {CD}}}^{{\rm {W}}}:{{\mathbb{R}}}^{M\times (3+1)}\times {{\mathbb{R}}}^{M^{\prime} \times (3+1)}\to {{\mathbb{R}}}^{+}$ as follows:

9 $$\begin{array}{l}{{\mathcal{D}}}_{{\rm {CD}}}^{\rm {{W}}}((Y,w),({Y}^{{\prime} },w^{\prime} )):= \frac{1}{N}\mathop{\sum }\limits_{i=1}^{M}{w}_{i}\mathop{\min }\limits_{{{\boldsymbol{c}}}^{{\prime} }\in {Y}^{{\prime} }}| | {Y}_{i}-{{\boldsymbol{c}}}^{{\prime} }| {| }_{2}^{2}\\\qquad\qquad\qquad\qquad\qquad\quad+\,\frac{1}{N^{\prime} }\mathop{\sum }\limits_{{i}^{{\prime} }=1}^{M^{\prime} }w^{{\prime} }_{i}\mathop{\min }\limits_{{\boldsymbol{c}}\in Y}| | {\boldsymbol{c}}-{Y}_{{i}^{{\prime} }}^{{\prime} }| {| }_{2}^{2}\,.\end{array}$$

Alternatively to the use of SD, we also try to mitigate the low-quality gradient issue by developing variants of CD that exploit information coming from the source and template surface normals. In fact, CD is agnostic of the closed surface structure of the manifold from which the points are sampled, as it solely relies on point-to-point distances. In particular, we consider two surface-aware corrections of CD. The first one—denoted as ${{\mathcal{D}}}_{{\rm {NCD}}}$ —simply consists of adding a regularization term that penalizes the discrepancy between the normals, i.e.

10 $$\begin{array}{rcl}{{\mathcal{D}}}_{{\rm {NCD}}}(Y,{Y}^{{\prime} })\!\!\!\!\!\!\!\!&&:= {{\mathcal{D}}}_{{\rm {CD}}}(Y,{Y}^{{\prime} })\,+\,\frac{{w}_{n}}{2M}\mathop{\sum }\limits_{i=1}^{M}{\left(1-{{\boldsymbol{n}}}_{{\boldsymbol{i}}}\cdot {{\boldsymbol{n}}}_{{{\boldsymbol{c}}}_{{\boldsymbol{i}}}^{{\prime} }}\right)}^{2}\\&&\,+\frac{{w}_{{n}}}{2M^{\prime} }\mathop{\sum }\limits_{{i}^{{\prime} }=1}^{M^{\prime} }{\left(1-{{\boldsymbol{n}}}_{{{\boldsymbol{i}}}^{{\prime} }}\cdot {{\boldsymbol{n}}}_{{{\boldsymbol{c}}}_{{\boldsymbol{i}}}^{{\prime} }}\right)}^{2}\,,\end{array}$$

where ${{{\boldsymbol{c}}}^{{\prime} }}_{i}:= \mathop{\min }\limits_{{{\boldsymbol{c}}}^{{\prime} }\in {Y}^{{\prime} }}| | {Y}_{i}-{{\boldsymbol{c}}}^{{\prime} }| {| }_{2}^{2}$, ${{\boldsymbol{c}}}_{{i}^{{\prime} }}:= \mathop{\min }\limits_{{\boldsymbol{c}}\in Y}| | {\boldsymbol{c}}-{{Y}^{{\prime} }}_{{i}^{{\prime} }}| {| }_{2}^{2}$, and ${w}_{{\rm {n}}}\in {{\mathbb{R}}}^{+}$ is a scale factor. Here ${{\boldsymbol{n}}}_{{\boldsymbol{i}}},{{\boldsymbol{n}}}_{{{\boldsymbol{i}}}^{{\prime} }},{{\boldsymbol{n}}}_{{{{\boldsymbol{c}}}^{{\prime} }}_{{\boldsymbol{i}}}},{{\boldsymbol{n}}}_{{{\boldsymbol{c}}}_{{{\boldsymbol{i}}}^{{\prime} }}}\in {{\mathbb{R}}}^{3}$ denote the (supposed known) outward unit normal vectors to the target surface, evaluated at ${Y}_{i},{Y}_{i}^{{\prime} },{{\boldsymbol{c}}}_{i}^{{\prime} },{{\boldsymbol{c}}}_{{i}^{{\prime} }}$, respectively. Also, ***a*** ⋅ ***b*** ≔ ∑_*j*_ ***a***_*j*_ ***b***_*j*_ is the standard inner product. Preliminary numerical tests suggested to set *w*_n_ = 10^−2^, which results in the normals’ penalization term to account for roughly 10% of the loss value. The second variant of CD, instead, is offered by the point-to-plane CD (PCD)^48^, denoted as ${{\mathcal{D}}}_{{\rm {PCD}}}$ and defined as

$${{\mathcal{D}}}_{{\rm {PCD}}}(Y,{Y}^{{\prime} }):= \frac{1}{N}\mathop{\sum }\limits_{i = 1}^{M}\mathop{\min }\limits_{{{\boldsymbol{c}}}^{{\prime} }\in {Y}^{{\prime} }}{\left(\left({Y}_{i}-{{\boldsymbol{c}}}^{{\prime} }\right)\cdot {{\boldsymbol{n}}}_{{\boldsymbol{i}}}\right)}^{2}+\frac{1}{N^{\prime} }\mathop{\sum }\limits_{{i}^{{\prime} } = 1}^{M^{\prime} }\mathop{\min }\limits_{{\boldsymbol{c}}\in Y}{\left(\left({\boldsymbol{c}}-{Y}_{i}^{{\prime} }\right)\cdot {{\boldsymbol{n}}}_{{{\boldsymbol{i}}}^{{\prime} }}\right)}^{2}\,,$$

where ${{\boldsymbol{n}}}_{{\boldsymbol{i}}},{{\boldsymbol{n}}}_{{\boldsymbol{i}}}^{{\prime} }$ are as in Eq. (10). The PCD computes the error projections along the normal directions, thus solely penalizing points that “move away” from the target local plane surface. For point clouds that are sampled from surfaces, this distance is better aligned with the perceived overlapping quality than the canonical CD. Analogously to ${{\mathcal{D}}}_{{\rm {CD}}}^{{\rm {W}}}$ defined in Eq. (9), weighted versions of ${{\mathcal{D}}}_{{\rm {NCD}}}$ and ${{\mathcal{D}}}_{{\rm {PCD}}}$, respectively denoted as ${{\mathcal{D}}}_{{\rm {NCD}}}^{{\rm {W}}}$ and ${{\mathcal{D}}}_{{\rm {PCD}}}^{{\rm {W}}}$, can be constructed.

#### Algorithm 1

AD-SVFD model training pipeline

1: **procedure** Train_AD_SVFD ($${{\mathcal{S}}}_{1},\ldots ,{{\mathcal{S}}}_{{N}^{{\rm {s}}}},{\mathcal{T}},E,B,M$$)

⊳ ${{\mathcal{S}}}_{i}$: *i*th source point cloud; ${\mathcal{T}}$: template point cloud; *E*: # epochs; *B*: batch size; *M*: # sampled points

2: Initialize ANN parameters ***Θ***

3: **for all**$i\in \{{i}_{1},\ldots ,{i}_{{N}^{\rm {{s}}}}\}$**do**

4: ${{\mathcal{L}}}_{i}^{{\rm {s}}},\,{{\mathcal{L}}}_{i}^{{\rm {t}}}\leftarrow {{\bf{0}}}_{M},\,{{\bf{0}}}_{M}$ ⊳ Initialize pointwise loss functions

5: Sample ${{\boldsymbol{z}}}_{i}^{{\rm {s}}} \sim {\mathcal{N}}\left({\bf{0}},\frac{2}{{N}_{z}}I\right)$ ⊳ Initialize shape code

6: $${{\mathcal{L}}}^{{\rm {t}}}\leftarrow {{\bf{0}}}_{M}$$

7: *e* ← 0

8: **while** *e* < *E***do** ⊳ Loop over epochs

9: $$b,\,{\mathcal{B}}\leftarrow 0,\,[\,]$$

10: **while**$b < \lceil \frac{{N}^{s}}{B}\rceil$**do** ⊳ Loop over batches

11: $\bar{B}\leftarrow B+1\,\,{\rm{if}}\,\,b < ({N}^{{\rm {s}}} \% B)\,\,{\rm{else}}\,\,B$ ⊳ Define batch size

12: Sample $${i}_{1},\ldots ,{i}_{\bar{B}} \sim {\mathcal{U}}(\{1,\ldots ,{N}^{{\rm {s}}}\}\setminus {\mathcal{B}})$$

13: ${{\mathcal{T}}}^{b}\leftarrow {\rm{PointSample}}({\mathcal{T}},{{\mathcal{L}}}^{t},M)$ ⊳ Sample template points

14: **for all**$i\in \{{i}_{1},\ldots ,{i}_{\bar{B}}\}$**do**

15: ${{\mathcal{S}}}_{i}^{{\rm {b}}}\leftarrow {\rm{PointSample}}({{\mathcal{S}}}_{i},{{\mathcal{L}}}_{i}^{{\rm {s}}},M)$ ⊳ Sample source points

16: ${\bar{{\boldsymbol{z}}}}_{i}\leftarrow {\rm{CodeSample}}({{\boldsymbol{z}}}_{i},{{\mathcal{S}}}_{i}^{{\rm {b}}})$ ⊳ Sample shape code

17: $${{\mathcal{S}}}_{i}^{{\rm {b}},(K)}\leftarrow {{\mathcal{D}}}_{{\rm {SVF}}}({{\mathcal{S}}}_{i}^{{\rm {b}}},{\bar{{\boldsymbol{z}}}}_{i},{\boldsymbol{\Theta }})$$ ⊳ Direct mapping

18: $${{\mathcal{T}}}_{i}^{\rm {{b}},(0)}\leftarrow {{\mathcal{I}}}_{{\rm {SVF}}}({{\mathcal{T}}}^{{\rm {b}}},{\bar{{\boldsymbol{z}}}}_{i},{\boldsymbol{\Theta }})$$ ⊳ Inverse mapping

19: ${{\mathcal{L}}}_{i}^{{\rm {s}}}\leftarrow {\mathcal{L}}({{\mathcal{S}}}_{i}^{{\rm {b}},(K)},{\mathcal{T}})$ ⊳ Direct mapping loss

20: ${{\mathcal{L}}}_{i}^{{\rm {t}}}\leftarrow {\mathcal{L}}({{\mathcal{T}}}_{i}^{\rm {{b}},(0)},{{\mathcal{S}}}_{i})$ ⊳ Inverse mapping loss

21: $${{\mathcal{L}}}_{{\rm {tot}}}\leftarrow \frac{1}{\bar{B}M}\mathop{\sum }\nolimits_{i,j = 1}^{\bar{B},M}({{\mathcal{L}}}_{i,j}^{{\rm {s}}}+{{\mathcal{L}}}_{i,j}^{{\rm {t}}})+{{\mathcal{L}}}_{{\rm {reg}}}$$ ⊳ Total loss

22: ${\boldsymbol{\Theta }}\leftarrow {\rm{Update}}\_{\rm{ANN}}({\boldsymbol{\Theta }},{{\mathcal{L}}}_{{\rm {tot}}})$ ⊳ Update ANN parameters

23: for all$i\in \{{i}_{1},\ldots ,{i}_{\bar{B}}\}$**do**

24: ${{\boldsymbol{z}}}_{i}\leftarrow {\rm {Update}}\_{\rm {Codes}}({{\boldsymbol{z}}}_{i},{{\mathcal{L}}}_{i}^{{\rm {s}}},{{\mathcal{L}}}_{i}^{{\rm {t}}})$ ⊳ Update shape codes

25: $$b,\,{\mathcal{B}}\leftarrow b+1,\,[{\mathcal{B}},{i}_{1},\ldots ,{i}_{\bar{B}}]$$

26 ${{\mathcal{L}}}^{{\rm {t}}}\leftarrow \frac{1}{{N}^{{\rm {s}}}}\mathop{\sum }\nolimits_{i = 1}^{{N}^{{\rm {s}}}}{{\mathcal{L}}}_{i}^{{\rm {t}}}$ ⊳ Average template loss

27: *e* ← *e* + 1

### Training procedure

The training pipeline of AD-SVFD is reported in detail in Algorithm 1. Hereafter, we only discuss a few relevant aspects. Algorithm 1 features a two-stage sampling procedure over the training epochs. Firstly, since we employ a batched stochastic optimization algorithm, we sample uniformly at random (without replacement) *B*-dimensional batches of source shapes with the associated shape codes (line 12). Then, for each of the selected point clouds, we sample *M*-dimensional sub-clouds (line 15); we also sample an *M*-dimensional sub-cloud for the template anatomy (line 13). In all tests, we set *B* = 8 and *M* = 2000. The motivation behind point clouds resampling is two-fold. On the one hand, it makes the training algorithm complexity independent of the level of refinement in the data, which is of paramount importance if the cardinality of the original clouds is large. Indeed, the complexity of all considered data attachment measures is quadratic in the number of points. On the other hand, resampling can be interpreted as a form of data augmentation and, as such, it allows improving robustness. Furthermore, we remark that the trilinear interpolation to compute the position-aware shape codes is repeated at every epoch (line 16), and it is also recurrently performed during time integration of the diffeomorphic flow ODE.

To further improve model performance, when using data attachment measures that allow for a pointwise evaluation (such as the ones based on CD), we implement a simple adaptive sampling procedure. This explains the presence of the template and source pointwise loss functions as input arguments to *PointSample* in lines 13 and 15, respectively. Specifically, at each training epoch, we sample ⌈(1−*a*)*M*⌉ points uniformly at random, whereas the remaining ⌊*aM*⌋ points are retained from the previous epoch, being the ones associated with the highest loss values. In this way, we oversample regions featuring larger mapping errors, tentatively driving the model towards homogeneously accurate predictions in space. In all tests, we consider *a* = 0.15, as resulting from the calibration procedure reported in Supplementary Note 3.

The joint optimization of the ANN parameters **Θ** (line 22) and of the latent shape codes ${\{{{\boldsymbol{z}}}_{i}\}}_{i = 1}^{{N}^{s}}$ (line 24) is achieved by minimizing the loss function reported in Eq. (4). Notably, to limit the kinetic energy of the system that connects the source to the target, thus encouraging minimal deformations and reducing the risk of overfitting, we introduce the regularization term ${{\mathcal{L}}}_{{\rm{reg}}}$:

11 $${{\mathcal{L}}}_{{\rm {reg}}}({\mathbf{\Theta }})\,:= \,\mathop{\sum }\limits_{i=1}^{{N}^{{\rm {s}}}}\mathop{\sum }\limits_{j=1}^{M}\mathop{\sum }\limits_{k=0}^{K-1}\left(\parallel {{\boldsymbol{v}}}_{i,j}^{{\rm {s}},(k)}({\mathbf{\Theta }}){\parallel }_{2}^{2}+\parallel {{\boldsymbol{v}}}_{i,j}^{{\rm {t}},(k+1)}({\mathbf{\Theta }}){\parallel }_{2}^{2}\right)\,,$$

where ${{\boldsymbol{v}}}_{i,j}^{s,(k)}$, ${{\boldsymbol{v}}}_{i,j}^{t,(k)}$ are defined as in Eqs. (5) and (7), respectively. For both **Θ** and the latent codes, we perform random initialization, drawing values from a Kaiming normal distribution^74^, and we rely on the first-order Adam optimizer^50^ for the update step. Hyperparameter calibration tests suggested adopting the same learning rate *λ* = 10^−3^ for all the trainable parameters. Finally, we run *E* = 500 training epochs, which guarantees convergence of the optimization procedure.

Two remarks are worth following. First, if the chosen data attachment measure does not allow for a pointwise evaluation, because it yields a cumulative discrepancy value, adaptive sampling cannot be performed. For instance, this is the case with SD. In Algorithm 1, the point sampling routines no longer depend on the loss at the previous epoch (lines 13,15), and no averaging over the points in the clouds is necessary to compute the total loss (line 21). Second, during inference, a pipeline similar to Algorithm 1, but much cheaper, is performed. Indeed, the optimization problem to be solved is much smaller, since just the *N*_*z*_ latent code entries associated with a single unseen shape have to be optimized. Remarkably, the low memory requirements enable the use of more advanced and memory-intensive optimizers, such as L-BFGS^51^, to fine-tune Adam predictions, attaining superlinear convergence rates.

## Supplementary information

Supplementary Information

## Data Availability

The dataset employed for the current study is publicly available at 10.5281/zenodo.15494901. All patient-specific anatomies are publicly available on the Vascular Model Repository (https://www.vascularmodel.com/dataset.html).The underlying code for this study is currently not available, but may be provided to qualified researchers upon request to the corresponding author. Future publications of the software are being considered to support transparency and reproducibility.

## Code availability

The underlying code for this study is currently not available, but may be provided to qualified researchers upon request to the corresponding author. Future publications of the software are being considered to support transparency and reproducibility.
