# Supplementary material for: Deformable registration and generative modelling of aortic anatomies by auto-decoders and neural ODEs
Source: NPJ Biol Phys Mech. 2025 Dec 3;2(1):26. doi: 10.1038/s44341-025-00029-z (PMC12675294; doi:10.1038/s44341-025-00029-z)
Supplement: Supplementary file 1 — Supplementary Information [file 44341_2025_29_MOESM1_ESM.pdf]

Deformable registration and generative modelling of aortic anatomies by auto–decoders and Neural ODEs

Supplementary Material

Riccardo Tenderini[1*], Luca Pegolotti[2,3,4], Fanwei Kong[3,4,5], Stefano Pagani[6], Francesco Regazzoni[6], Alison L. Marsden[2,3,4,7], Simone Deparis[1]

[1*]Institute of Mathematics, EPFL, Lausanne, Switzerland.
[2]Department of Bioengineering, Stanford University, CA, USA.
[3]Department of Pediatrics, Stanford University, CA, USA.
[4]Institute for Computational and Mathematical Engineering, Stanford University, CA, USA.
[5]Department of Mechanical Engineering and Materials Science, Washington University, St. Louis, MO, USA.
[6]MOX – Department of Mathematics, Politecnico di Milano, Piazza Leonardo da Vinci 32, Milan, Italy.
[7]Cardiovascular Institute, Stanford University, CA, USA.

*Corresponding author(s). E-mail(s): riccardo.tenderini@outlook.com;

This file includes:

- Supplementary Note 1–4
- Supplementary Figure 1
- Supplementary Table 1–4
- Supplementary Algorithm 1

Supplementary Note 1: Deformable registration by thin–plate spline interpolation

We consider thin–plate splines (TPS), a special case of polyharmonic splines introduced in [1], that admits a natural radial basis function representation via the infinite–support kernel function $\kappa(x) = x^2 \log x$. Let us consider a pair of geometries (G_α, G_β). Let us suppose to know $M \in \mathbb{N}$ exact point–to–point correspondences $\{(\boldsymbol{x}_\alpha^j, \boldsymbol{x}_\beta^j)\}_{j=1}^M$. Then, TPS interpolation finds a diffeomorphism that deforms G_α into G_β by solving the following energy minimization problem:

$$\begin{aligned} \vec{g}_\star = \; & \underset{\vec{g} \in \mathcal{G}}{\arg\min} \sum_{j=1}^{M} \|\boldsymbol{x}_\beta^j - \vec{g}(\boldsymbol{x}_\alpha^j)\|_2^2 \; + \; w_H \|H_g(\boldsymbol{x}_\alpha^j)\|_F^2 \;, \\ & \text{where} \quad \mathcal{G} := \left\{ \vec{g} : \mathbb{R}^3 \to \mathbb{R}^3 : \; \vec{g}(\vec{x}) = \sum_{j=1}^{M} g_j \; \kappa\left(\|\vec{x} - \boldsymbol{x}_\alpha^j\|_2\right) \right\} . \end{aligned} \tag{1}$$

Here, $H_g : \mathbb{R}^3 \to \mathbb{R}^{3\times 3}$ denotes the Hessian of g, and $\|\cdot\|_F : \mathbb{R}^3 \to \mathbb{R}^+$ is the Frobenius norm operator. The smoothing parameter $w_H \in \mathbb{R}^+$ allows to balance the goodness of fit with the regularity of the deformation. The most relevant limiting factor to the use of TPS interpolation is the availability of reliable point–to–point correspondences.

In this work, we identify corresponding points by exploiting the peculiar structure of the geometries at hand. Indeed, vascular anatomies consist of the intersection of several vessels, each one featuring a tube–like shape. In *SimVascular* [2], vessels are conveniently modelled by their centerline, which is approximated by a trivariate cubic spline, and by a number of surface contours, planar closed lines that define the cross–sectional vessel lumen boundary at selected locations along the centerline. Even if *SimVascular* allows to accurately describe surface contours using B–splines, we relied on a much simpler approximation, supposing the cross–sectional areas to be circular and centered at the centerline points. Furthermore, to derive more precise point–to–point correspondences, we partitioned some of the vessel into chunks, which are defined depending on the location of eventual branches. Indeed, as displayed in Figure 2 (b) in the manuscript, each anatomy in the dataset features $N_v = 5$ vessels (aorta, LSA, RSA, RCCA, LCCA), but $N_p = 7$ vessel portions (AA, DA, BA, LSA, RSA, LCCA, RCCA). Now, let $M_p \in \mathbb{N}$ be the total number of points sampled in each vessel portion, and let $M_c \in \mathbb{N}$ be the number of points sampled at each contour. In this work, we consider $M_p = 250$ and $M_c = 4$. For a given vessel portion p of G_α, the sampled points $\{\boldsymbol{x}_{\alpha,p}^j\}$ are structured as follows:

- $M_p/(M_c + 1)$ points are uniformly distributed along the centerline;
- $(M_c M_p)/(M_c+1)$ points are uniformly distributed along the (approximated) circular contours, corresponding to each sampled centerline point.

The final set of sampled points is then given by $X_\alpha = \bigcup_{\ell=1}^{N_p} \{\boldsymbol{x}_{\alpha,p_\ell}^j\}_{j=1}^{M_p}$. The same sampling strategy is used to define the set of sampled points X_β for G_β.

For every point in a child branch, we compute the convex hull generated by its $1,000$ nearest neighbours in the parent vessel. If the point belongs to the convex hull, it means that it lies inside the parent vessel and so it is removed from the dataset. Additionally, also the points that lie outside of the convex hull by a distance smaller than τD_p are discarded, where $D_p \in \mathbb{R}^+$ is the maximal distance between two points in the child branch and $\tau \in \mathbb{R}^+$ is a prescribed threshold. This helps in guaranteeing the well–posedness of the TPS interpolation problem. Ultimately, the total number of points sampled at a vessel portion is $\widetilde{M}_p \leq M_p$. If a point is removed from X_α, the corresponding one is removed from X_β, and viceversa.

While the correspondences quality for the centerline points is often remarkable, the same consideration does not hold for the ones sampled on the lateral surface. Indeed, since the centerline is an open curve, the knowledge of the curvilinear coordinates alone is sufficient to derive solid correspondences. However, the surface contours are planar closed curves; this entails that reliably corresponding samples can be selected only upon convenient choices of two–dimensional reference frames. In fact, the selection of matching surface samples closely depends on the identification of topologically equivalent zero–degree angles in the cross–sectional planes. To this aim, we employ the Bishop frame of reference [3, 4], a coordinates system for curves, which is defined by transporting a given reference frame (forward and/or backward) along the curve itself. Two peculiarities of the Bishop frame are noteworthy. Firstly, one of its vectors always coincides with the curve tangent. Secondly, the coordinates system exhibits a uniform zero twist along the curve. Therefore, if we are able to define equivalent reference frames for the cross–sectional planes located at two corresponding centerline points, then such frames can be robustly "extended" to the whole vessel. In this work, the equivalent reference frames have been derived using *ad hoc* techniques, based on the relative positions of inlets and outlets. For instance, the vector that defines the zero–degree angle at the aorta's inlet contour is computed as the orthogonal projection of the vector that connects the aorta's centerline endpoints.

In order to guarantee the quality of point–to–point correspondences, an initial rigid alignment of the geometries is crucial. To this aim, we employ the Coherent Point Drift (CPD) algorithm, a point set registration method based on Gaussian Mixture Models [5]. Compared to the most popular Iterative Closest Point (ICP) algorithm [6] and to its most widely employed variants and alternatives (such as Levenberg–Marquardt ICP [7] or Robust Point Matching [8, 9]), CPD proved to be more accurate and robust in presence of noise, outliers and missing points. Nonetheless, CPD is an iterative algorithm, and hence its accuracy is strictly linked to the choice of a good initial guess. For this reason, prior to the execution of CPD, we perform the following three–steps *ad hoc* rigid registration and rescaling procedure, as reported at line 6 in Algorithm 1.

Let $\mathcal{S}_\alpha$, $\mathcal{S}_\beta$ be two point clouds, computed from the surface meshes $\mathcal{M}_\alpha$, $\mathcal{M}_\beta$. Furthermore, let us suppose to know the position of the aorta's inlet center and the normal vector to the aorta's outlet, for both geometries. Then, we proceed as follows:

1. *Rescaling*: translate $\mathcal{S}_\alpha$, so that its barycenter coincides with the one of $\mathcal{S}_\beta$. Perform an isotropic rescaling of $\mathcal{S}_\alpha$, so that the maximal distance between points in $\mathcal{S}_\alpha$ equals the one in $\mathcal{S}_\beta$. We call the output $\mathcal{S}_\alpha^{(1)}$.
2. *Translation*: translate $\mathcal{S}_\alpha^{(1)}$, so that its aorta's inlet center coincides with the one of $\mathcal{S}_\beta$. We call the output $\mathcal{S}_\alpha^{(2)}$.
3. *Rotation*: rotate $\mathcal{S}_\alpha^{(2)}$ at the aorta's inlet center around the aorta's outlet normal vector by the angle ϑ that minimizes the Chamfer Distance (CD) between $\mathcal{S}_\beta$ and $\mathcal{S}_\alpha^{(2)}$. We call the output $\bar{\mathcal{S}}_\alpha$, which serves as the initial guess for the CPD iterations.

Table 1 reports the results of the TPS interpolation algorithm, with and without prior rigid registration, obtained on two of the patients in the dataset (*P#090*, *P#272*) and averaged over all the shapes in the dataset (see Figure 2 in the manuscript), except from *P#091*, that serves as reference. The pointwise registration errors, computed at all the cell centers of the available surface triangulations, are quantifies through the forward and backward local distances (FLD and BLD), expressed in cm. The former identifies the distance of each point in the mapped geometry from the closest one in the target, while the latter is the distance of each point in the target from the closest one in the mapped geometry. Figure 1 offers a visualization of the results, showing the positions of the interpolation points and the pointwise FLD values. A few considerations deserve attention. Firstly, TPS interpolation attains a notable degree of accuracy, with FLDs that are always well below the 1 cm threshold. Secondly, preliminary rigid registration is crucial when the original orientation of the target geometry differs from the reference one. This is showcased by patient *P#272*; indeed, the geometry obtained upon TPS interpolation without rigid registration is extremely irregular and convoluted, particularly in the aortic arch. Finally, we underline that TPS interpolation is rather sensitive to the values of (*i*) the smoothing parameter w_H in Eq.(1), and (*ii*) the tolerance τ. The calibration of the latter is particularly important to obtain good quality results. In the reported tests, we select $\tau = 5 \cdot 10^{-3}$ for *P#090*, and $\tau = 2.5 \cdot 10^{-3}$ for *P#272*. However, to compute the aggregate metrics in Table 1, we set $\tau = 5 \cdot 10^{-3}$ for all the geometries; this justifies why average errors are larger than patient–specific ones.

Supplementary Note 2: Data augmentation pipeline

The proposed thin–plate spline (TPS) interpolation algorithm can provide good quality mapping results at a contained computational cost. However, robustness is a major drawback. Indeed, undesired artifacts are often introduced for too small values of w_H (and for inadequate choices for τ), while large values of w_H negatively impact the overall goodness of fit. For this reason, we do not use TPS interpolation to solve the deformable registration problem on the vascular anatomies at hand, but we nonetheless exploit it for data augmentation.

Our TPS–based data augmentation pipeline is reported in Algorithm 1. The procedure involves the evaluation of "partial" TPS interpolators, where the word "partial" refers to the fact that only points from a subset of randomly selected vessel portions are considered. More specifically, at each iteration, we choose a random pair of geometries from the source cohort (line 5), whose corresponding sampled point sets are X_α, X_β, and a random number of vessel portions $L \in \{1, 2\}$ (line 8). Firstly, we rigidly deform the points in X_α that belong to the selected vessel portions; this leads to the definition of $\bar{X}_\alpha = \bigcup_{\ell=1}^{L} \{\bar{X}_{\alpha,p_\ell}\}$ (lines 6,7). Then, the interpolation values are computed as follows (line 12):

$$\tilde{X}_\beta := \bigcup_{\ell=1}^{L} \left\{(1 - C_\ell)\bar{X}_{\alpha,p_\ell} + C_\ell X_{\beta,p_\ell}\right\} \ , \quad \text{with} \quad C_\ell \sim \mathcal{U}\left([0.5, 1]\right) \ .$$

Hence, the points selected from X_α are not mapped to the corresponding ones in X_β, but to some intermediate locations along the connecting segments, whose precise position depends on the random matching factors C_ℓ. The derived TPS interpolator is used to deform the surface mesh $\mathcal{M}_\alpha$ of the first shape, so that a new triangulation $\mathcal{M}'$ is generated (lines 13,14). Finally, the resulting geometry is added to the dataset if the quality of the associated surface mesh is sufficiently high (line 15). Specifically, we require the scaled Jacobian — the determinant of the Jacobian divided by the product of the two longest edges — to be strictly positive for all cells and to have a bottom decile average value greater than 0.1. From a qualitative point of view, this choice allows to obtain "trustworthy" geometries that do not feature undesired artifacts and irregularities. Incidentally, we remark that the obtained surface meshes are not used to perform numerical simulations, but only serve as a tool for shape discretization. Therefore, it is not necessary to require a high level of regularity, and we can accept the presence of a few bad elements.

Figure 2 (c) in the manuscript displays some of the shapes obtained by deforming the anatomies of four different patients with the proposed data augmentation pipeline. Despite being relatively simple, we remark the ability of the method to generate rather diverse shapes. Using Algorithm 1, we created 50 new geometries from each of the "original" anatomies, hence assembling a dataset comprising $1,020$ shapes. However,

the final dataset used to train and test the AD–SVFD model only counts 902 geometries (88.4%). The remaining 118 ones have been manually removed, since they were showing artifacts that could not be captured with the implemented mesh quality check.

Supplementary Note 3: hyperparameters calibration

We focus on the calibration of the most relevant hyperparameters of AD–SVFD.

ANN hyperparameters tuning

At first, we fine–tune the hyperparameters that are not related to the implicit neural representation of the source shapes. Since the latent codes are not involved in the calibration procedure, we can consider the case of a single shape–to–shape registration. This choice allows to dramatically lighten and speedup the training (from ≈ 8 h to ≈ 5 min), hence enabling an exhaustive exploration of the hyperparameters' space at affordable computational costs.

We consider ten hyperparameters, namely: the activation function, width and depth of FA–NN (ϕ_{FA}, W_{FA}, L_{FA}) and DF–NN (ϕ_{DF}, W_{DF}, L_{DF}), the refinement level of the FPE (N_e), the penalty term w_v, the learning rate $\lambda = \lambda_\Theta$, and the adaptive sampling factor a. To limit the number of trainings and yet retain an extensive coverage of the hyperparameters' space, we run the Tree–structured Parzen Estimator (TPE) Bayesian algorithm [10] for five different shapes (*P#090*, *P#144*, *P#188*, *P#207*, *P#272*) Considering quantized values, the total number of possible hyperparameters combinations is $8.64M$. However, adopting TPE, we only perform 500 trainings for each target shape; hence the overall duration of the fine–tuning procedure sets to ≈ 30 h per patient.

In order to identify a common (sub–)optimal set of hyperparameters, we marginalize the results of the five TPE runs with respect to the hyperparameter values. Firstly, for every patient, we associate every model with a score $s \in \mathbb{R}^+$, computed by averaging the mean forward and backward local distances associated with the direct and inverse mapping. To balance the contributions of the five patients, we normalize the model score s by the best (i.e. the lowest) score s^*; this defines the normalized score $\tilde{s}$. Then, for each patient, every hyperparameter value is associated with the bottom decile average with respect to $\tilde{s}$, computed considering all the trained models that feature such value. Finally, for each hyperparameter value, we compute an aggregate performance score $S \in \mathbb{R}^+$ by averaging the bottom decile averages obtained on the five patients. Table 2 reports the results of the calibration procedure.

Even though the set of hyperparameters reported in Table 2 features (sub–)optimal properties for single shape–to–shape registration, those are not guaranteed to automatically transfer to the "complete" AD–SVFD model. In fact, with this configuration, the training of AD–SVFD fails, since all shape codes converge to the zero vector, leading to large errors. Empirically, we found that the problem is related to vanishing gradient issues in the trainable feature augmentation model compartment FA–NN. To circumvent this pitfall, we changed the FA–NN activation function ϕ_{FA} from *ReLU* to *leaky–ReLU* (with negative slope equal to 0.2); this allowed to retain remarkable accuracy levels even in the multiple–shape scenario.

Shape code hyperparameters tuning

We focus on the calibration of two hyperparameters related to the shape codes, namely the regularization factor w_z (see Eq.(4)) and the learning rate λ_z. Table 3 reports the maximal pointwise errors — quantified through the forward and backward local distances FLD and BLD, in cm — corresponding to different choices of w_z (for $\lambda_z = 10^{-3}$) and λ_z (for $w_z = 10^{-3}$). Concerning the regularization parameter, the results show little sensitivity, provided that sufficiently small values are considered. Indeed, all the models featuring $w_z \leq 10^{-3}$ yield similar results, but accuracy deteriorates for larger values. Conversely, the quality of the results heavily depends on the choice of the learning rate λ_z. Indeed, sensibly larger errors are obtained when either too small (e.g. $\lambda_z = 10^{-4}$) or too large (e.g. $\lambda_z = 10^{-2}$) values are selected. Ultimately, based on the obtained results, we set $w_z = 10^{-3}$ and $\lambda_z = 10^{-3}$. Notably, we choose w_z as large as possible, in order to maximally regularize the latent space without compromising the registration accuracy.

Supplementary Note 4: robustness assessment

To evaluate the robustness of AD–SVFD in greater detail, Table 4 reports the maximal pointwise errors — expressed by average between FLD and BLD for both the direct (LD–D) and inverse (LD–I) map — obtained with our approach on ten testing geometries, one per cross–validation fold. The results are compared to those achieved by three single–shape registration models, namely LDDMM [11], I–ResNet–LDDMM [12], and SVFD (i.e. our approach lacking the auto–decoder structure). We further indicate the average and standard deviation of all methods on both error metrics and the average inference time. It should be emphasized that a cross–validation procedure was not feasible for the competing methods, since they are all inherently designed for single shape–to–shape registration tasks.

The obtained results highlight the good generalization capability of AD–SVFD. Although its average accuracy is slightly lower than that of LDDMM (+5% and +16% on LD–D and LD–I) and I–ResNet–LDDMM (+22% and +5% on LD–D and LD–I), this drawback is largely offset by a substantial reduction of the inference time (−85% compared to LDDMM, from 10min to 1.5min, and −81% compared to I–ResNet–LDDMM, from 8min to 1.5min). Moreover, we note that the SVFD model outperforms the two competing single–shape registration approaches, achieving 56% and 33% lower LD–D and LD–I compared to LDDMM, and 39% and 49% lower LD–D and LD–I compared to I–ResNet–LDDMM. Additionally, it provides accurate predictions even for the "critical" patients *P#205* and *P#207*. These findings confirm that the use of latent shape codes, albeit enabling faster inference and generative applications, inevitably entails accuracy losses and performance pitfalls, largely attributable to data scarcity.

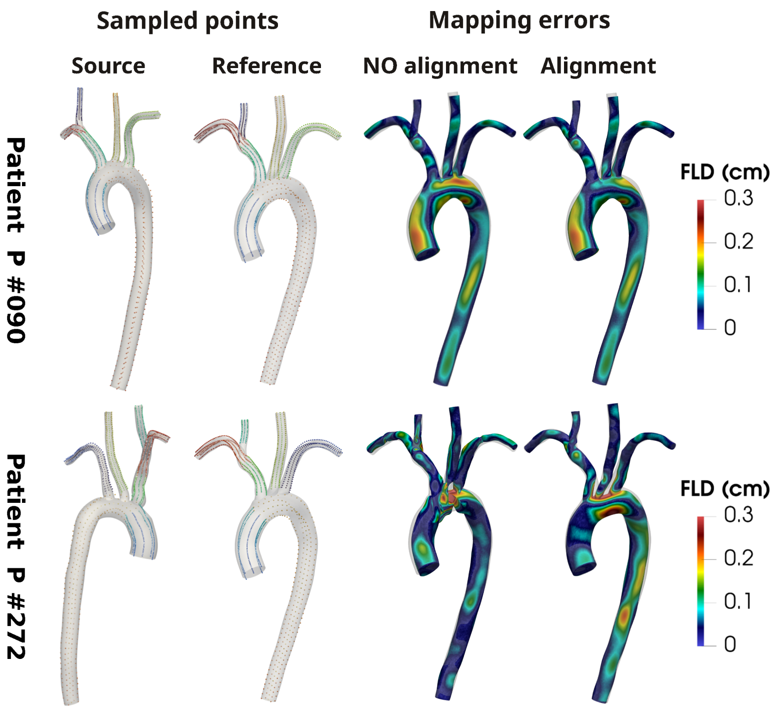

Supplementary Figure 1: *Visualization of the TPS interpolation results. In particular, for two patients in the dataset (P#090 and P#272) we show the locations of the interpolation points in the target and template geometries — color–coded so that corresponding points share the same value — and the pointwise mapping errors, quantified through the forward local distance (FLD), expressed in* cm. *For the mapping results, we compare the errors obtained without and with a preliminary rigid registration of the geometries to the template by the Coherent Point Drift algorithm. For reference, the template inlet diameter is* 1.31 cm *for* P#091.

Supplementary Table 1: *TPS deformable registration errors. In particular, we report the average and maximal pointwise errors for the registration of two of the patients in the dataset to the template and the average errors over all patients. Patient P#091 serves as reference and it is not considered in the average errors calculation. In all cases, we compare the results obtained without and with a preliminary rigid registration of the geometries to the template by the Coherent Point Drift algorithm. The errors are quantified through the forward and backward local distances (FLD and BLD), expressed in* cm. *For reference, the template inlet diameter is* 1.31 cm *for* P#091.

		Max Errors		Avg Errors	
		**FCD**	**BCD**	**FCD**	**BCD**
**Without Rigid Alignment**	***P#090***	0.2918	0.0777	0.2615	0.0770
	P#272	0.5760	0.0670	0.3428	0.0641
	Average	0.7304	0.0861	0.4480	0.0759
**With Rigid Alignment**	***P#090***	0.2786	0.0736	0.2349	0.0739
	P#272	0.4667	0.0759	0.3002	0.0744
	Average	0.6211	0.0825	0.3908	0.0756

Supplementary Table 2: *Results of the AD–SVFD hyperparameters calibration procedure. To save computational resources, we worked in a single shape–to–shape registration scenario and employed the Tree–structured Parzen Estimator algorithm, considering five different shapes. We refer to the text for a detailed definition of each hyperparameter. Every hyperparameter value is associated with the aggregate performance score S, computed from the average pointwise forward and backward local distances related to the direct and inverse mappings. Low values of S correspond to accurate models. The optimal hyperparameter choices are marked in green. The yellow cells denote the hyperparameter values that were changed when incorporating the shape codes, for the simultaneous registration of multiple shapes. Notation:* l–ReLU *stands for* leaky–ReLU*, with a negative slope equal to* 0.2.

Parameter						
ϕ_{FA}	***ReLU*** 1.0425	***l − ReLU*** 1.0655	***ELU*** 1.0586	***SELU*** 1.0573		
ϕ_{DF}	***ReLU*** 1.0632	***l − ReLU*** 1.0488	***ELU*** 1.0578	***SELU*** 1.0522		
W_{FA}	2^3 1.0502	2^4 1.0548	2^5 1.0531	2^6 1.0481	2^7 1.0758	
W_{DF}	2^6 1.0677	2^7 1.0489	2^8 1.0413	2^9 1.0715		
L_{FA}	**0** 1.0632	**1** 1.0627	**2** 1.0557	**3** 1.0474	**4** 1.0629	
L_{DF}	**4** 1.0489	**5** 1.0444	**6** 1.0504	**7** 1.0709	**8** 1.0717	
N_e	**0** 1.0532	**1** 1.0639	**2** 1.0515	**3** 1.0336	**4** 1.0393	**5** 1.0604
w_v	10^{-6} 1.0579	10^{-5} 1.0371	10^{-4} 1.0554	10^{-3} 1.0654	10^{-2} 1.0815	
λ_Θ	10^{-4} 1.1698	$10^{-3.5}$ 1.0671	10^{-3} 1.0272	$10^{-2.5}$ 1.0565	10^{-2} 1.1083	
a	**0.00** 1.0830	**0.05** 1.0535	**0.10** 1.0542	**0.15** 1.0404	**0.20** 1.0737	**0.25** 1.0738

Supplementary Table 3: *Registration results of AD–SVFD considering different values of the regularization parameter w_z and of the shape codes learning rate λ_z. In particular, we report the maximal pointwise errors on training and testing datapoints, obtained for six different values of w_z and for five different values of λ_z. The errors are quantified through the forward and backward local distances (FLD and BLD), expressed in* cm*. The best value for each performance metric is marked with a rectangular box. For reference, the template shape inlet diameter is* 1.31 cm*, while the average inlet diameter in the dataset is* 1.45 cm.

	**Train errors (in cm)**				**Test errors (in cm)**			
	**Direct**		**Inverse**		**Direct**		**Inverse**	
$\boldsymbol{w_z}$	**FLD**	**BLD**	**FLD**	**BLD**	**FLD**	**BLD**	**FLD**	**BLD**
**0.0**	[0.2095]	0.2201	[0.2583]	[0.2257]	0.2725	[0.1934]	[0.2422]	0.2860
$\mathbf{10^{-5}}$	0.2333	0.2308	0.2834	0.2398	[0.2714]	0.2092	0.2564	0.2905
$\mathbf{10^{-4}}$	0.2166	0.2207	0.2719	0.2259	0.2853	0.2238	0.2815	0.3187
[$\mathbf{10^{-3}}$]	0.2162	[0.2175]	0.2686	0.2297	0.2777	0.2253	0.2562	[0.2642]
$\mathbf{10^{-2}}$	0.2413	0.2353	0.3078	0.2408	0.3149	0.2751	0.3099	0.3713
$\mathbf{10^{-1}}$	0.3019	0.2794	0.3855	0.3020	0.4587	0.3641	0.4294	0.6546
$\boldsymbol{\lambda_z}$	**FLD**	**BLD**	**FLD**	**BLD**	**FLD**	**BLD**	**FLD**	**BLD**
$\mathbf{10^{-4}}$	0.2557	0.2404	0.3107	0.2680	0.4529	0.2679	0.3105	0.4771
$\mathbf{5 \cdot 10^{-4}}$	[0.2072]	0.2176	[0.2665]	[0.2176]	0.2783	[0.2144]	0.2671	0.3117
[$\mathbf{10^{-3}}$]	0.2162	[0.2175]	0.2686	0.2297	[0.2777]	0.2253	[0.2562]	[0.2642]
$\mathbf{5 \cdot 10^{-3}}$	0.2574	0.2444	0.2938	0.2572	0.3556	0.2264	0.2893	0.3202
$\mathbf{10^{-2}}$	1.1374	2.1948	2.2775	1.1860	1.5228	1.8003	1.8297	1.5442

Supplementary Table 4: *Performances of AD–SVFD on testing geometries, as resulting from cross–validation experiments, compared to three alternative single–shape registration methods. For ten patients, one per cross–validation fold, we compare the maximal pointwise errors obtained by AD–SVFD with the ones of LDDMM [11], I–ResNet–LDDMM [12], and SVFD, namely our shape registration model without the auto–decoder structure. The methods' performances are quantified by the direct and inverse local distances (LD–D and LD–I), both expressed in* cm *and computed as the average of the maximal forward and backward LD for the direct and inverse mapping, respectively. For each method, we also report the average and standard deviation of the two error metrics and the average inference time, computed across the ten patients. The best results for single–shape models are marked with rectangular boxes; critical geometries identifiers are underlined. For reference, the template shape inlet diameter is* 1.31 cm*, while the average inlet diameter in the dataset is* 1.45 cm*.*

P #	Max Errors (in cm)							
	LDDMM		I–ResNet		SVFD		AD–SVFD	
	**LD–D**	**LD–I**	**LD–D**	**LD–I**	**LD–D**	**LD–I**	**LD–D**	**LD–I**
**90**	0.1520	0.2979	0.2108	0.2303	[0.1409]	[0.1667]	0.2365	0.2842
**94**	0.1582	0.2144	0.2992	0.1690	[0.1350]	[0.1036]	0.2500	0.2748
**142**	0.3193	[0.2461]	0.3478	0.4095	[0.1408]	0.2776	0.3245	0.3904
**143**	[0.1477]	0.2090	0.3106	0.3199	0.1517	[0.1589]	0.2548	0.2534
**187**	0.2994	0.2340	0.2994	0.2972	[0.1561]	[0.1554]	0.3438	0.3808
**201**	0.1483	[0.2021]	0.3221	0.4348	[0.1323]	0.2293	0.2646	0.2961
**<u>205</u>**	1.1109	0.2381	0.3484	0.3026	[0.1604]	[0.1965]	0.6460	0.4533
**<u>207</u>**	0.8325	0.2916	0.3058	0.3271	[0.1799]	[0.1981]	0.7910	0.3240
**277**	[0.1295]	0.3878	0.2493	0.3362	0.1644	[0.1962]	0.2514	0.3438
**278**	0.1481	0.5301	0.2652	0.3177	[0.1479]	[0.2254]	0.2652	0.3177
**Avg**	0.3454	0.2851	0.2959	0.3144	0.1509	0.1908	0.3628	0.3319
**Std**	0.3478	0.0974	0.0410	0.0727	0.0140	0.0456	0.1837	0.0583
**Time**	10 min		8 min		12 min		1.5 min	

Supplementary Algorithm 1 TPS–based data augmentation

procedure AugmentDataset(N, $\mathcal{M}_1, \ldots, \mathcal{M}_G$, $X_1, \ldots, X_G$)
  ▷ N: number of geometries; $\mathcal{M}_k$: k–th mesh; X_k: k–th set of sampling points

  $n \leftarrow 0$
  $\mathcal{D} \leftarrow [\mathcal{M}_1, \ldots, \mathcal{M}_G]$ ▷ Initialize the dataset

  while $n < N$ **do**
    Sample $\alpha, \beta \sim \mathcal{U}(\{1, \ldots, G\}),\ \alpha \neq \beta$ ▷ Select two shapes
    Ad hoc rigid registration of $\mathcal{M}_\alpha$ to $\mathcal{M}_\beta$
    CPD–based rigid registration of $\mathcal{M}_\alpha$ to $\mathcal{M}_\beta$

    Sample $L \sim \mathcal{U}(\{1, 2\})$ ▷ Select vessel portions number
    Sample L vessel portions $p_1, \ldots, p_L$ ▷ Select vessel portions
    Sample $C_\ell \sim \mathcal{U}([0.5, 1])$, $\ell \in \{1, \ldots, L\}$ ▷ Select matching factors

    $\tilde{X}_\alpha \leftarrow \{X_{\alpha,p_\ell}\}_{\ell=1}^{L}$ ▷ Interpolation points
    $\tilde{X}_\beta \leftarrow \{\{(1 - C_\ell)X_{\alpha,p_\ell} + C_\ell X_{\beta,p_\ell}\}\}_{\ell=1}^{L}$ ▷ Interpolation field
    $\mathcal{I} \leftarrow$ TPS_interpolator($\tilde{X}_\alpha, \tilde{X}_\beta$)
    $\mathcal{M}' \leftarrow \mathcal{I}(\mathcal{M}_\alpha)$ ▷ Query the interpolator

    if quality($\mathcal{M}'$) is good **then** ▷ Check mesh quality
      $\mathcal{D} \leftarrow [\mathcal{D}, \mathcal{M}']$ ▷ Update dataset
      $n \leftarrow n + 1$

  return $\mathcal{D}$

Supplementary References

[1] Duchon, J. Splines minimizing rotation–invariant semi–norms in Sobolev spaces. *Constructive Theory of Functions of Several Variables: Proceedings of a Conference Held at Oberwolfach April 25–May 1, 1976*, 85–100 (Springer, 1977).

[2] Updegrove, A. *et al.* SimVascular: an open source pipeline for cardiovascular simulation. *Annals of biomedical engineering* **45**, 525–541 (2017).

[3] Bishop, R. L. There is more than one way to frame a curve. *The American Mathematical Monthly* **82**, 246–251 (1975).

[4] Ebrahimi, M., Butscher, A. & Cheong, H. A low order, torsion deformable spatial beam element based on the absolute nodal coordinate formulation and Bishop frame. *Multibody System Dynamics* **51**, 247–278 (2021).

[5] Myronenko, A. & Song, X. Point set registration: coherent point drift. *IEEE transactions on pattern analysis and machine intelligence* **32**, 2262–2275 (2010).

[6] Besl, P. J. & McKay, N. D. Method for registration of 3D shapes. *Sensor fusion IV: control paradigms and data structures*, Vol. 1611, 586–606 (Spie, 1992).

[7] Fitzgibbon, A. W. Robust registration of 2D and 3D point sets. *Image and vision computing* **21**, 1145–1153 (2003).

[8] Gold, S., Rangarajan, A., Lu, C.-P., Pappu, S. & Mjolsness, E. New algorithms for 2D and 3D point matching: pose estimation and correspondence. *Pattern recognition* **31**, 1019–1031 (1998).

[9] Chui, H. & Rangarajan, A. A new algorithm for non–rigid point matching. *Proceedings IEEE Conference on Computer Vision and Pattern Recognition. CVPR 2000 (Cat. No. PR00662)*, Vol. 2, 44–51 (IEEE, 2000).

[10] Bergstra, J., Bardenet, R., Bengio, Y. & Kégl, B. Algorithms for hyper–parameter optimization. *Advances in neural information processing systems* **24** (2011).

[11] Bône, A., Louis, M., Martin, B. & Durrleman, S. Deformetrica 4: an open–source software for statistical shape analysis. *Shape in Medical Imaging: International Workshop, ShapeMI 2018, Held in Conjunction with MICCAI 2018, Granada, Spain, September 20, 2018, Proceedings*, 3–13 (Springer, 2018).

[12] Amor, B. B., Arguillère, S. & Shao, L. ResNet–LDDMM: advancing the LDDMM framework using deep residual networks. *IEEE Transactions on Pattern Analysis and Machine Intelligence* **45**, 3707–3720 (2022).
